# Molecular mechanisms of seed dormancy release by gas plasma-activated water technology

**DOI:** 10.1093/jxb/erac150

**Published:** 2022-04-15

**Authors:** Giles Grainge, Kazumi Nakabayashi, Tina Steinbrecher, Sue Kennedy, Junchen Ren, Felipe Iza, Gerhard Leubner-Metzger

**Affiliations:** Department of Biological Sciences, Royal Holloway University of London, Egham, Surrey TW20 0EX, UK; Department of Biological Sciences, Royal Holloway University of London, Egham, Surrey TW20 0EX, UK; Department of Biological Sciences, Royal Holloway University of London, Egham, Surrey TW20 0EX, UK; Elsoms Seeds Ltd, Spalding, Lincolnshire PE11 1QG, UK; Wolfson School of Mechanical, Electrical and Manufacturing Engineering, Loughborough University, Leicestershire LE11 3TU, UK; Wolfson School of Mechanical, Electrical and Manufacturing Engineering, Loughborough University, Leicestershire LE11 3TU, UK; Division of Advanced Nuclear Engineering, Pohang University of Science and Technology (POSTECH), Pohang, Gyeongbuk 790-784, South Korea; Department of Biological Sciences, Royal Holloway University of London, Egham, Surrey TW20 0EX, UK; Laboratory of Growth Regulators, Institute of Experimental Botany, Czech Academy of Sciences and Faculty of Science, Palacký University Olomouc, CZ-78371 Olomouc, Czech Republic; University of Birmingham, UK

**Keywords:** Abscisic acid metabolism, *Arabidopsis thaliana*, endosperm weakening, gas plasma-activated water, nitrogen signalling, non-thermal atmospheric gas plasma technology, plant hormone signalling, reactive oxygen species, seed dormancy

## Abstract

Developing innovative agri-technologies is essential for the sustainable intensification of global food production. Seed dormancy is an adaptive trait which defines the environmental conditions in which the seed is able to germinate. Dormancy release requires sensing and integration of multiple environmental signals, a complex process which may be mimicked by seed treatment technologies. Here, we reveal molecular mechanisms by which non-thermal (cold) atmospheric gas plasma-activated water (GPAW) releases the physiological seed dormancy of *Arabidopsis thaliana*. GPAW triggered dormancy release by synergistic interaction between plasma-generated reactive chemical species (NO_3_^–^, H_2_O_2_, ·NO, and ·OH) and multiple signalling pathways targeting gibberellin and abscisic acid (ABA) metabolism and the expression of downstream cell wall-remodelling genes. Direct chemical action of GPAW on cell walls resulted in premature biomechanical endosperm weakening. The germination responses of dormancy signalling (*nlp8*, *prt6*, and *dog1*) and ABA metabolism (*cyp707a2*) mutants varied with GPAW composition. GPAW removes seed dormancy blocks by triggering multiple molecular signalling pathways combined with direct chemical tissue weakening to permit seed germination. Gas plasma technologies therefore improve seed quality by mimicking permissive environments in which sensing and integration of multiple signals lead to dormancy release and germination.

## Teaser

Gas plasma-activated water agri-technologies mimick the multitude of environmental signals to enhance seed germination and growth required for the sustainable intensification of food production.

## Introduction

‘Plasma agriculture’ is a rapidly emerging field in which pre-planting and post-harvest applications using non-thermal atmospheric gas plasma are developed into environment-friendly agri-technologies for the sustainable production of food (Bourke *et al.*, 2018; Ito *et al.*, 2018; Ranieri *et al.*, 2021). Seeds are the beginning (sowing) and the end (harvesting) of many food chains important to human existence. High-quality crop seeds are the delivery systems of technological advances in plant breeding and seed treatment to agriculture and food chains (Finch-Savage and Leubner-Metzger, 2006; Nonogaki, 2017; Yan *et al.*, 2014). Gas plasma is often defined as ‘the fourth state of matter’ due to its high energetic state. In non-thermal (cold, non-equilibrium) atmospheric plasma, reactive species including free radicals such as reactive oxygen species (ROS) and reactive nitrogen species (RNS) are produced at ambient temperature and atmospheric pressure (Graves, 2012; Park *et al.*, 2013; Lukes *et al.*, 2014; Takamatsu *et al.*, 2014; Lu *et al.*, 2016; Zhou *et al.*, 2020; Ranieri *et al.*, 2021). The formation of specific reactive species is dependent on the gas admixture, flow of the gas, plasma frequency, and temperature. The non-thermal plasma’s gaseous phase has demonstrated its potential as an effective method for decontaminating surfaces of heat-sensitive food products and to plant seeds by inactivating microbial pathogens (Bourke *et al.*, 2018; Ito *et al.*, 2018; Stepanova *et al.*, 2018). During these gas-phase treatments of dry seeds, effects on germination performance were also noted and attributed to direct physical changes (etching) of the seed coat surface resulting in improved wetting, permeability, and water uptake (Bormashenko *et al.*, 2015; Ling *et al.*, 2015; Zhou *et al.*, 2016; Bafoil *et al.*, 2019). Progress in applying these as innovative sustainable seed technologies requires knowledge about the underpinning molecular mechanisms, as well as a clear distinction between the target seed quality traits dormancy, germination, and subsequent seedling growth.

A technological application distinct from using it in the ‘gaseous’ form is to utilize plasma chemistry for the treatment of biological materials through ‘activating’ water. Gas plasma-activated water (GPAW) is produced by a gas plasma discharge at the gas–liquid interface (Lukes *et al.*, 2014; Takamatsu *et al.*, 2014; Bruggeman *et al.*, 2016; Liu *et al.*, 2019; Zhou *et al.*, 2020). In the case of air as carrier gas, this initiates the formation of transient ROS and RNS such as hydroxyl (·OH) and nitric oxide (·NO) radicals, which react to form more stable compounds such as hydrogen peroxide (H_2_O_2_), nitrite (NO_2_^–^), and nitrate (NO_3_^–^). Treatment of non-dormant seeds with GPAW enhanced their germination speed as well as the growth of the emerged seedlings (Park *et al.*, 2013; Zhou *et al.*, 2016; Bafoil *et al.*, 2018; Liu *et al.*, 2019). Works with *Arabidopsis thaliana* demonstrated that GPAW increased the permeability of the seed coat (testa) and slightly promoted testa rupture of non-dormant wild type (Col-0) seeds (Bafoil *et al*., 2018, 2019). However, neither were the chemical species in the GPAW which cause these effects identified, nor were the underpinning molecular mechanisms studied in these works with non-dormant *A. thaliana* seeds. Seed dormancy is an innate seed property that defines the environmental conditions in which a seed is able to germinate (Finch-Savage and Leubner-Metzger, 2006). Several well-distinguished seed dormancy classes are known and include the physiological dormancy of *A. thaliana*. The molecular mechanisms of physiological dormancy induction during seed maturation on the mother plant and its release after shedding are well known (Nambara *et al.*, 2010; Graeber *et al.*, 2012; Yan *et al.*, 2014; Shu *et al.*, 2016; Nonogaki, 2017; Steinbrecher and Leubner-Metzger, 2017; Duermeyer *et al.*, 2018; Bailly, 2019), but it is not known if GPAW can release physiological dormancy of mature seeds of any species. Due to their larger size and similar seed structure, the seeds of *Lepidium sativum*, a Brassicaceae relative of Arabidopsis, provide a model system for the direct biomechanical measurement of endosperm weakening (Müller *et al.*, 2009; Lee *et al.*, 2012; Graeber *et al.*, 2014; Steinbrecher and Leubner-Metzger, 2017).

In this study we compared the effects of GPAW (produced in a bubble reactor; Supplementary Fig. S1) treatment on physiologically dormant and after-ripened (non-dormant) *A. thaliana* seeds. We demonstrate that depending on the type of GPAW used, defined RNS and ROS in GPAW caused the dormancy release and stimulated germination via altering the metabolism and signalling of the antagonistically acting hormones abscisic acid (ABA) and gibberellins (GAs). We found that GPAW altered the expression of the associated genes, and mutant analysis revealed the signalling pathways involved. Further to this, biochemical and biomechanical analysis demonstrated that GPAW promoted micropylar endosperm (cap) weakening by direct and indirect mechanisms. Therefore, GPAW technology targets mechanisms conserved among plants, opening up an enticing prospect for the agricultural industry and the sustainable intensification of food production.

## Materials and methods

### Seed material and germination assays

Plant materials used in this study were *A. thaliana* (L.) Heynh. C24, Col-0, and mutants *cyp707a2-1* (Kushiro *et al.*, 2004), *dog1-2* (Nakabayashi *et al.*, 2012), *nlp8-2* (Yan *et al.*, 2016), and *prt6-1* (Holman *et al.*, 2009). The *nlp8-2* mutant was kindly provided by Eiji Nambara (University of Toronto, Canada). The *prt6-1* mutant (SAIL 1278_H11) was obtained from the Nottingham Arabidopsis Stock Centre, UK. All the mutants were in the Col-0 background. All plants were grown at 20 °C (16/8 h day/night cycle) until flowering, and were then transferred to 16 °C (16/8 h day/night cycle) to establish higher primary dormancy as previously described (Nakabayashi *et al.*, 2012) except C24 which remained at 20 °C. Post-harvest, seeds were dried for 2 d on silica gel (15% relative humidity) before being stored at –20 °C to maintain primary dormancy. Seeds were defrosted at room temperature 1 h prior to assays. For after-ripening, seeds were stored at 21 °C and 33% relative humidity for up to 6 weeks. For germination assays, ~60 seeds in triplicate were imbibed in 600 µl of deionized water (dH_2_O; water purifier system Select Purewater 300, Purite Ltd, Trevose, PA, USA) and autoclaved water (control) or the specified GPAW (45 min discharged) within a 6 cm Petri dish with a single filter paper (MN 713, Macherey-Nagel, Düren, Germany), incubated in the growth chamber set at 20 °C in constant white light (~100 µmol m^–2^ s^–1^) (MLR-352 Environmental Test Chamber, Panasonic, Bracknell, UK). Radicle emergence was scored and germination percentage was graphed with the mean ±SEM and statistically compared through ANOVA and Tukey’s analysis using Prism 7.01 software (GraphPad Software, Inc., USA). *Lepidium sativum* L. FR14 seeds (‘Keimsprossen’, Juliwa) (Scheler *et al.*, 2015) were propagated, and harvested seeds were dried under 15% relative humidity before storage at –20 °C until used in the experiment.

### Dielectric barrier discharge gas plasma-activated water (GPAW) production

The plasma reactor engineered to produce GPAW (Supplementary Fig. S1) consists of 12 high voltage AC electrodes covered in a dielectric material fixed below a gas-permeable stainless-steel membrane (Wright *et al.*, 2018). Above the membrane is a tank containing 100 ml of dH_2_O. A carrier gas (air or He–O_2_ admixture) flows past the electrodes at 1 SLPM (standard litre per minute), and then through the membrane and H_2_O. For activation, plasma is formed between the electrodes and the membrane within the carrier gas. The non-thermal atmospheric gas plasma after-glow then flows through the membrane bubbling up through the water, facilitating radical and ion diffusion into the water. To produce Air-GPAW, air was used as carrier gas with the plasma sustained at 15.8 kV and 27.1 kHz. To produce He/O_2_-GPAW, a helium (98%) and oxygen (2%) mixture was used as carrier gas with the plasma sustained at 8.5 kV and 29.3 kHz. In both cases, the plasma was modulated with an on-time of 100 ms and a duty cycle of 30%. Compressed air (UN1002, BOC Ltd, Guildford, UK), helium (UN1046, N4.6, BOC Ltd), and oxygen (UN1072, N5.0, BOC Ltd) gases were used for all treatments, and both their mixture and flow rate were controlled through Alicat MC-series mass flow controllers (Alicat Scientific, Tucson, AZ, USA). Voltage and frequency measurements were recorded using a Tektronix P6015A high voltage probe (Tektronix, OR, USA) and a TBS1102B digital oscilloscope (Tektronix, USA).

### GPAW chemical analysis

H_2_O_2_ was quantified colorimetrically using the titanium sulfate method (Eisenberg, 1943). The peroxotitanium (IV) complex formed by the reaction of H_2_O_2_ with titanyl ions under acidic conditions was quantified by measuring the absorbance at 407 nm. A standard curve constructed using H_2_O_2_ solution (30%, Sigma-Aldrich, MO, USA) was used to calculate a molar extinction coefficient. NO_2_^–^ and NO_3_^–^ were quantified simultaneously using Griess and vanadium (III) chloride (VCl_3_) reagents in an assay described in detail by Garcia-Robledo *et al.* (2014). ·OH radicals were quantified through the hydroxylation of the chemical probe terephthalic acid; the resultant 2-hydroxy terephthalic acid (HTA) is fluorescent (excitation 315 nm, and emission at 425 nm). The ·OH radical synthetic rate was quantified during both plasma discharge and post-discharge. Standards of HTA (Sigma-Aldrich) were used for quantification and conversion to molar units (Sahni and Locke, 2006). For measurement during discharge, 100 ml of 2 mM HTA dissolved in 10 mM phosphate buffer (pH 6.8) was placed in the reactor chamber, and fluorescence was recorded from 300 µl samples at timed intervals. For post-discharge measurements, GPAW samples were removed from the reactor and combined with double concentration HTA reagent (4 mM HTA, 20 mM phosphate buffer, pH 6.8) in a 1:1 ratio, total volume 2 ml, sealed in a 6 cm Petri dish, incubated at 21 °C in constant light, and 300 µl samples were removed at timed intervals. For measurement, samples were placed in UV-transparent 96-well plates (UV transparent, Costar® 3635, Corning Inc., NY, USA) and fluorescence was measured (excitation 315 nm, and emission at 425 nm) using a Spark™ Multimode Plate Reader (Tecan Group Ltd, Männedorf, Switzerland).

### RNA extraction, cDNA synthesis, and RT–qPCR analysis

For each sample, 10 mg of whole seeds were collected at the specified times, frozen in liquid nitrogen, and stored at –80 °C. RNA extraction was performed using the cetyltrimethylammonium bromide (CTAB) method (Chang *et al.*, 1993) with the following modifications. Chloroform extraction was repeated three times before LiCl precipitation, additionally repeated three times following dissolving the RNA in SSTE buffer, and extracted RNA was treated with DNase (Qiagen, Manchester, UK) according to the manufacturer’s instructions. RNA quantity and purity were measured using a Spark™ Multimode Plate Reader (Tecan Group Ltd), and only samples with absorbance ratios of at least 2.0 (260/280 nm) and 2 (260/230 nm) were used for cDNA synthesis. Quantitative reverse transcription–PCR (RT–qPCR) analysis was conducted as described (Graeber *et al.*, 2011). In brief, cDNA was synthesized with random pentadecamers from 1 µg of total RNA using Superscript III reverse transcriptase (Invitrogen, Paisley, UK) in a 20 µl volume and diluted 20-fold. qPCR was performed with ABsolute qPCR SYBR mix (Thermo Fisher Scientific, Oxford, UK) on a Biorad CFX96 system (Bio-Rad Laboratories, Watford, UK) with 140 nM gene-specific primer sets (Supplementary Table S1). The PCR program was as follows: 15 min at 95 °C, followed by 50 cycles of 15 s at 95 °C, 30 s at 60 °C or 64 °C, and 30 s at 72 °C, and then melt curve analysis was performed. Two technical replicates were performed on five biological replicates for each sample. The expression values were normalized against the geometric mean of two reference genes, *HBT* (At2g20000) and *TIP41-Like* (At4g34270), and relative expression values were shown as fold change against the indicated samples using the 2^–ΔΔCt^ method (Livak and Schmittgen, 2001; Graeber *et al.*, 2011).

### Biomechanical analysis

Puncture force measurements of *L. sativum* FR14 micropylar endosperm (cap) tissue was conducted using a custom-made machine as described earlier (Lee *et al.*, 2012; Graeber *et al.*, 2014; Steinbrecher and Leubner-Metzger, 2017). Seeds were imbibed for 1 h in 6 cm Petri dishes with two filter papers (4007130050 Macherey-Nagel) and 1.5 ml of H_2_O in constant light at 20 °C. The 1 h imbibed seeds were then dissected in moist conditions to obtain the micropylar endosperm (cap) tissues. The isolated caps were then incubated for 2 h in 1.5 ml of treatment solution [control (dH_2_O), Air-GPAW, or He/O_2_-GPAW] in constant light at 20 °C. The 3 h treated caps were then fixed into a metal mould before a metal probe (diameter 0.3 mm) was driven (0.7 mm min^–1^) through the caps. The force it took to rupture the caps was recorded with the displacement. The cap puncture force (tissue resistance) was determined as the maximal force from the displacement–force curve, and the cap tissue elasticity was calculated as the slope of the linear portion of the displacement-force curve (Fig. 5).

### Xyloglucan endotransglycosylase (XET) enzyme activity assays

*Lepidium sativum* FR14 seeds were imbibed in dH_2_O and incubated as described above. Cap tissues were prepared from 1, 3, and 10 h imbibed seeds for monitoring the XET activities in the caps in germination processes. Another set of cap tissues dissected from 1 h imbibed seeds (isolated cap) were further incubated for 2 h in 1.5 ml of treatment solution (water for the control, Air-GPAW, or He/O_2_-GPAW) in constant light at 20 °C for monitoring direct effects of GPAW on caps. Total protein was extracted from 22 caps per sample and the XET enzyme activities were assayed using the method of Fry (1997) with the modifications described in Holloway *et al.* (2021) except for the following changes. All protein samples were adjusted to 2 µg µl^–1^, 10 µg protein samples were used for each reaction, and were incubated in darkness at 20 °C for 4 h. Matrices were measured dry, twice, before native protein loading (fluorescence *t*=0) and post-incubation plus washing and drying (fluorescence *t*=4 h). The percentage of transglycosylated XLLG–SR (sulforhodamine-labelled xyloglucan nonasaccharide) relative to the xyloglucan substrate, after blank subtraction (extraction buffer loaded matrix), was used as the relative XET activity value.

## Results

### GPAW interferes with the seed hormone metabolism to release physiological dormancy and promote germination

**Fig. 1.**
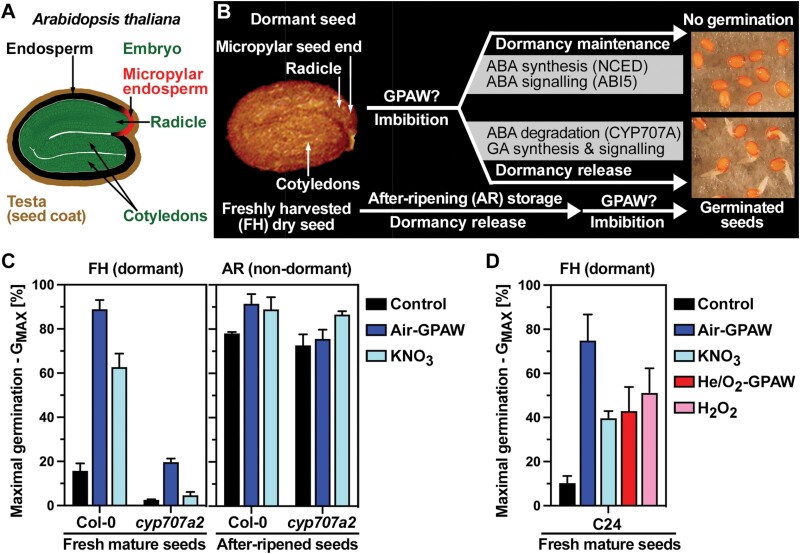
Seed dormancy release by gas plasma-activated water (GPAW). (A) Drawing of a mature *Arabidopsis thaliana* seed. (B) Visualization of *A. thaliana* seed states and dormancy release pathways. The freshly harvested (FH) dry seed has physiological dormancy, which can be released either during imbibition, such as by cold stratification, or by after-ripening (AR) storage. Abscisic acid (ABA) and gibberellin (GA) metabolism and signalling differ between dormancy maintenance and release to control germination in response to the environment. (C) The release of physiological dormancy of FH *A. thaliana* Col-0 seeds by treatment with Air-GPAW involves ABA degradation via the *CYP707A2* gene. Air-GPAW treatment of AR seeds does not significantly improve the maximal germination (G_MAX_) of either wild-type or *cyp707a2* mutant seeds. (D) The effects of Air-GPAW, He/O_2_-GPAW, 5 mM KNO_3_, and 300 µM H_2_O_2_ on the G_MAX_ of FH (dormant) *A. thaliana* C24 seeds. Discharge times of 45 min were used for the GPAW production (Supplementary Fig. S1). Means ±SEM are presented for G_MAX_.

Freshly harvested (FH) mature seeds of *A. thaliana* (Fig. 1A) have physiological dormancy which means that they do not germinate when imbibed under favourable conditions (Graeber *et al.*, 2012). The dormancy maintenance in imbibed FH seeds is achieved, at least in part, by enhanced ABA biosynthesis and signalling (Fig. 1B). In agreement with this, FH *A. thaliana* Col-0 and C24 seed populations were dormant in our experiments when imbibed in water (control). The maximum germination percentages (G_MAX_) remained low at 10–15% even after prolonged incubation (Fig. 1C, D). In contrast to water, imbibition in Air-GPAW caused dormancy release and resulted in high G_MAX_ values of 80–90% (Fig. 1C, D). He/O_2_-GPAW also caused dormancy release of a fraction of the seed population, resulting in a G_MAX_ of ~40% (Fig. 1D). The release of physiological seed dormancy in the imbibed state by cold stratification is known to be achieved by increased GA biosynthesis (Ogawa *et al.*, 2003) and ABA degradation via ABA 8ʹ-hydroxlase encoded by the *CYP707A2* gene (Kushiro *et al.*, 2004). In agreement with a key role for CYP707A2 in the GPAW-mediated dormancy release, Air-GPAW treatment of non-germinating (FH) *cyp707a2* mutant seeds did not result in full dormancy release as observed in the wild type (Fig. 1C). Release of physiological dormancy can also be achieved in the dry state during after-ripening (AR) storage (Fig. 1B). Non-dormant (AR) *A. thaliana* seed populations fully germinated when imbibed in water (control) with no or only small G_MAX_ increases upon GPAW treatment (Fig. 1C). In contrast to the small effect on AR seed, the finding that GPAW treatment released the physiological dormancy of FH seeds resulting in a large increase in G_MAX_ (Fig. 1) was intriguing and triggered follow-up experiments to identify the dormancy-releasing compounds in GPAW and to investigate the underpinning molecular mechanisms.

**Fig. 2.**
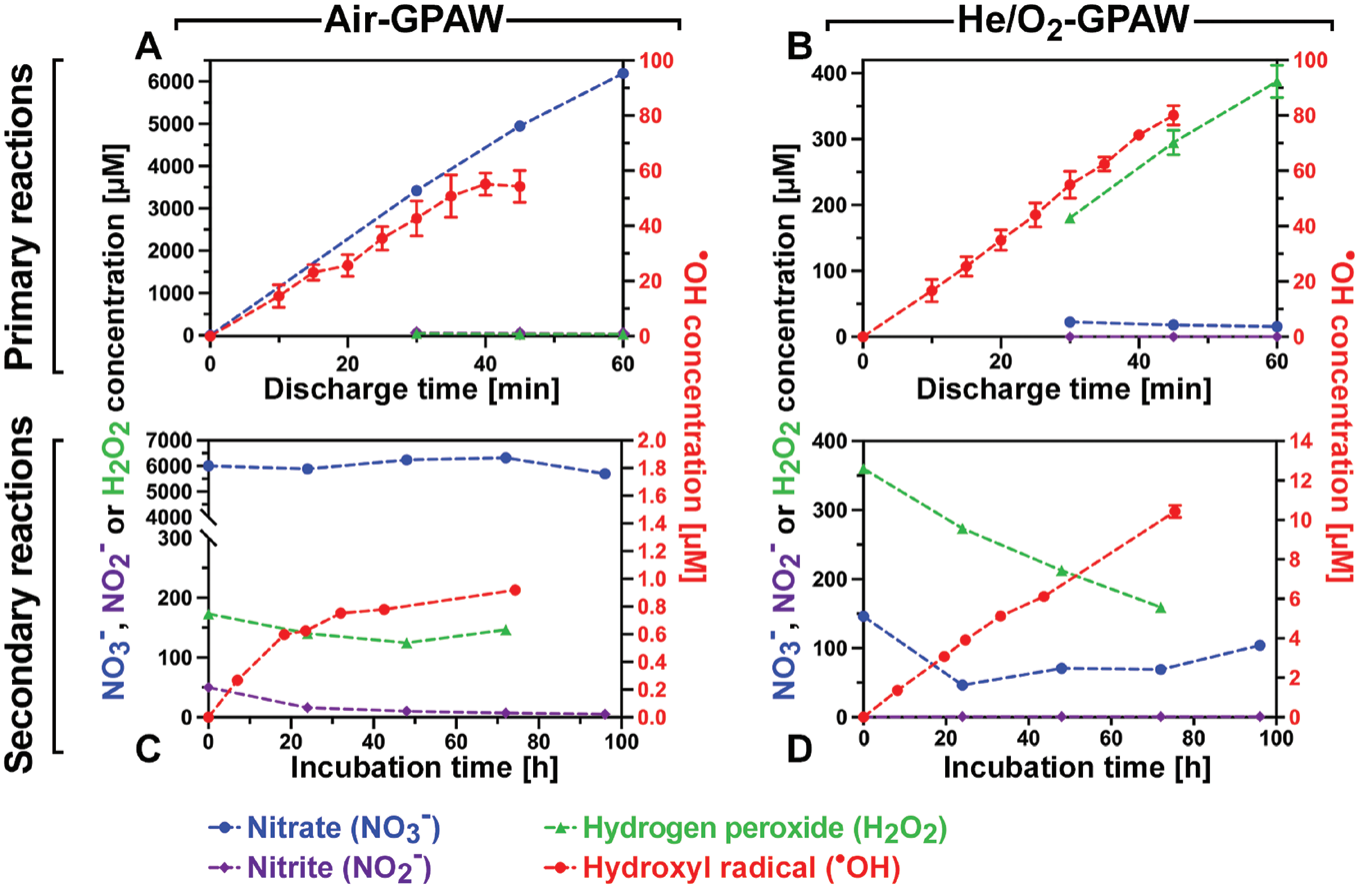
Chemical characterization of gas plasma-activated water (GPAW). (A) Air-GPAW. (B) He/O_2_-GPAW. Top panels: time evolution of the concentrations of major chemical species produced in GPAW during the discharge treatment in the plasma bubble reactor; primary reactions (Supplementary Fig. S1). Bottom panels: secondary chemical reactions after removal from the bubble reactor. Quantification of major chemical species as a function of the incubation time at ~22 °C after the plasma treatment. Note that Air-GPAW and He/O_2_-GPAW differ considerably in their composition; means ±SEM are presented.

The GPAW produced for this study was standardized using a bubble reactor (Supplementary Fig. S1), and the active species generated under two preparation regimes, Air-GPAW and He/O_2_-GPAW, were quantified (Fig. 2). As the water was exposed to plasma during GPAW production, primary reactions led to an increase of the concentration of nitrate (NO_3_^–^) in Air-GPAW (Fig. 2A) and, as expected, negligible nitrate production in He/O_2_-GPAW (Fig. 2B). H_2_O_2_ increased to reach 388 µM in He/O_2_-GPAW (Fig. 2B) and was >10-fold lower in Air-GPAW (Fig. 2A). The highly reactive hydroxyl radical (·OH) increased to ~60 µM steady-state concentration in both Air-GPAW (Fig. 2A) and He/O_2_-GPAW (Fig. 2B), while nitrite (NO_2_^–^) was produced in Air-GPAW and absent in He/O_2_-GPAW. The two types of GPAW, therefore, shared ·OH production, but differed in that NO_3_^–^ accumulated over discharge time only in Air-GPAW and H_2_O_2_ only in He/O_2_-GPAW. Post-discharge, the concentration of active species in the water evolved over time due to secondary reactions. This included initially a sharp drop in the very short-lived ·OH radical (half-life 1 ns), followed by secondary ·OH steady-state production. During a GPAW incubation period of up to 100 h, the concentration of H_2_O_2_ remained at 100–300 µM in both GPAWs (Fig. 2C,D). However, the two GPAWs differed in that NO_3_^–^ remained high at ~6 mM, and ·OH secondary production was very slow (0.01 µM h^–1^), leading to <1 µM ·OH concentrations in Air-GPAW (Fig. 2C), while in He/O_2_-GPAW NO_3_^–^ remained very low and a >10-fold faster (0.14 µM h^–1^) ·OH secondary production led to >10 µM ·OH concentrations. The observed seed dormancy release with GPAW (Fig. 1) may therefore be achieved by NO_3_^–^, H_2_O_2_, ·OH (Fig. 2), and other ROS/RNS derived from these (including ·NO radicals; Supplementary Fig. S2) produced *in vitro* (Lukes *et al.*, 2014; Takamatsu *et al.*, 2014; Zhou *et al.*, 2020) and/or *in planta* (Müller *et al.*, 2009; Albertos *et al.*, 2015; Kolbert *et al.*, 2019).

### GPAW-mediated dormancy release involves signalling of reactive species to induce genes for GA biosynthesis, ABA degradation, and cell wall remodelling

**Fig. 3.**
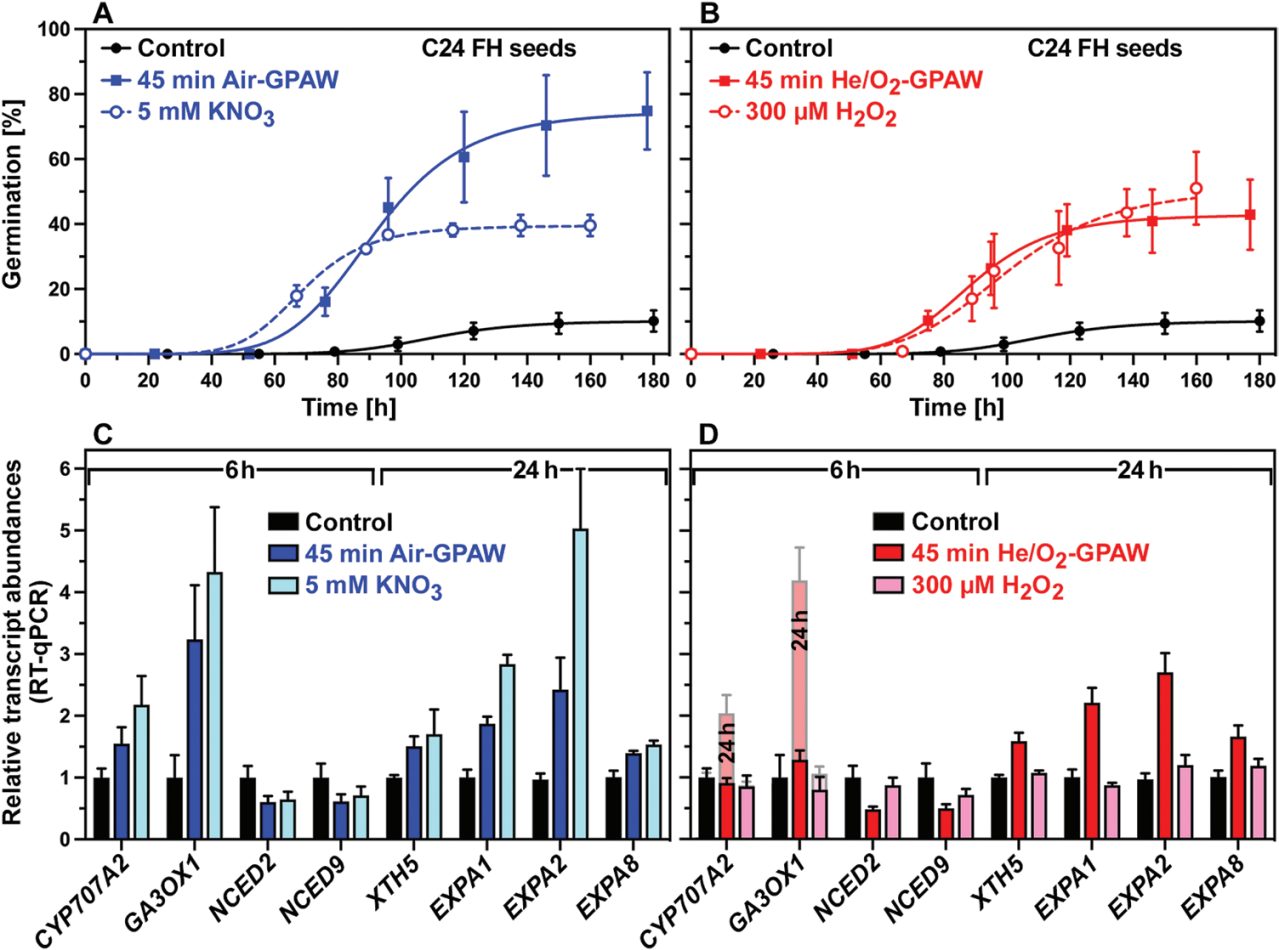
GPAW-induced gene expression associated with dormancy release and germination. (A) Dormancy release of freshly harvested (FH) *Arabidopsis thaliana* C24 seeds by Air-GPAW (45 min discharge time) and 5 mM KNO_3_ mimicking the Air-GPAW’s NO_3_^–^ concentration (see Fig. 2A) as compared with the control (dH_2_O). (B) Dormancy release of FH C24 seeds by He/O_2_-GPAW (45 min discharge time) and 300 µM H_2_O_2_ mimicking the He/O_2_-GPAW’s H_2_O_2_ concentration (see Fig. 2B). (C) RT–qPCR analyses of seed transcript abundances at 6 h for key genes encoding enzymes for ABA degradation (*CYP707A2*), GA biosynthesis (*GA3OX1*), ABA biosynthesis (*NECD2/9*), and at 24 h for CWRP genes known to be involved in endosperm weakening and germination. Relative mean ±SEM values for the Air-GPAW and NO_3_^–^ treatments are compared with the 6 h and 24 h samples of the control series (set to 1 for each gene). (D) RT–qPCR analyses of seed transcript abundances for the He/O_2_-GPAW and H_2_O_2_ treatments. Relative mean ±SEM values compared with the 6 h and 24 h controls are presented.

Figure 3 shows that GPAW-mediated dormancy release caused changes in the expression patterns of key genes in GA and ABA metabolism, as well as in downstream genes encoding cell wall-remodelling proteins (CWRPs) known to be required for germination (Finch-Savage and Leubner-Metzger, 2006; Nambara *et al.*, 2010; Graeber *et al.*, 2012). The full dormancy release of imbibed FH C24 seeds by Air-GPAW (Fig. 3A) was associated with the very early (6 h) up-regulation of *GA 3-oxidase 1* (*GA3OX1*; biosynthesis of bioactive GA) and *CYP707A2* (ABA degradation) and down-regulation of 9-*cis*-epoxycarotenoid dioxygenase *NCED2* and *NCED9* (ABA biosynthesis) transcript abundances (Fig. 3C). The partial dormancy release of imbibed FH C24 seeds by He/O_2_-GPAW (Fig. 3B) was also associated with the down-regulation of *NCED2* and *NCED9* at 6 h, but the up-regulation of *GA3OX1* and *CYP707A2* was slower and became evident only at 24 h (Fig. 3D). From studies of dormant and non-dormant *A. thaliana* seeds, it is known that these key genes are regulated early during imbibition to control the GA/ABA balance (Nakabayashi *et al.*, 2005; Bethke *et al.*, 2007; Preston *et al.*, 2009; Liu *et al.*, 2010; Graeber *et al.*, 2014), which is decisive in the control of germination by dormancy maintenance or release (Fig. 1B). Nitrate treatment resulted in very similar expression patterns to Air-GPAW (Fig. 3C), suggesting that nitrate signalling may be involved in the dormancy release by the ~5 mM NO_3_^–^ in the Air-GPAW (Fig. 2A). The dormancy release caused by nitrate treatment was, however, only partial (G_MAX_ ~40%; Fig. 3A), suggesting that in addition ·OH (Fig. 2) and other ROS and RNS pathways may be involved (Supplementary Fig. S2) to achieve the full dormancy release.

**Fig. 4.**
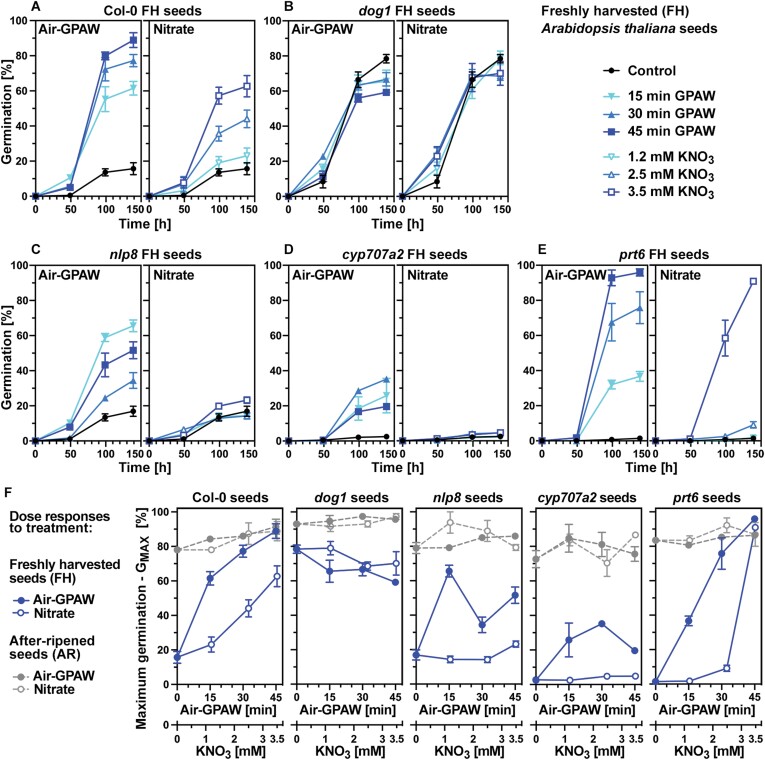
Germination responses of *Arabidopsis thaliana* mutant seeds to Air-GPAW treatment. (A) Germination responses of freshly harvested (FH) *A. thaliana* Col-0 seeds to Air-GPAW with increasing discharge time and the corresponding NO_3_^–^ mimic with increasing concentration. (B) Germination responses of FH *dog1* mutant seeds. (C) Germination responses of FH *nlp8* mutant seeds. (D) Germination responses of FH *cyp707a2* mutant seeds. (E) Germination responses of FH *prt6* mutant seeds. (F) Dose responses to Air-GPAW and the corresponding NO_3_^–^ mimic for the maximum germination (G_MAX_) of FH compared with AR wild-type and mutant seeds. Mean ±SEM germination values over time of seed populations incubated in constant white light at 20 °C are presented.

In agreement with this, He/O_2_-GPAW treatment caused partial dormancy release associated with similar but slower changes in the *GA3OX1*, *CYP707A2*, and *NCED2/9* transcript abundances (Fig. 3). The ~10-fold lower nitrate concentration in He/O_2_-GPAW is too low to alone trigger the dormancy release (Fig. 4), but ·OH, H_2_O_2_ (Fig. 2), or other ROS signalling molecules may be involved. It is known that high H_2_O_2_ concentrations (5–10 mM) fully release *A. thaliana* dormancy in association with very early up-regulation of *GA3OX1* and *CYP707A2* expression in imbibed seeds (Liu *et al.*, 2010). In contrast to this, low H_2_O_2_ concentrations [<1 mM (Liu *et al.*, 2010) or 300 µM (Fig. 3B)] are less effective. In addition, the treatments induced the expression of CWRP genes such as those encoding expansins and xyloglucan endotransglycolases/hydrolase (XTHs; *XTH5*, *EXPA1*, *EXPA2*, and *EXPA8*) responsible for endosperm weakening (Fig. 3C,D). Taken together, these findings demonstrate that GPAW treatment alters the expression of GA and ABA metabolism genes, and suggest that the expected change in the GA/ABA balance and resulting downstream CWRP gene expression may cause the GPAW-mediated dormancy release. To further investigate which of the known major ROS and RNS signalling pathways (Supplementary Fig. S2) are involved in these GPAW responses, we utilized specific *A. thaliana* mutants in dose–response experiments (Fig. 4).

### Mutant analysis reveals that GPAW acts to release dormancy via several key signalling pathways involved in the control of germination by physiological dormancy

Increased Air-GPAW discharge time leading to elevated production of major chemical species (Fig. 2) resulted in a dose-dependent increase in physiological dormancy release of FH *A. thaliana* seeds (Fig. 4A). This GPAW dose response resulted in a partial dormancy release (G_MAX_ ~60%) for the 15 min discharge time to full dormancy release (G_MAX_ ~90%) for the 45 min discharge time. A dose-dependent response was also evident for nitrate, which reached partial dormancy release (G_MAX_ ~60%) at 3.5 mM KNO_3_ in FH Col-0 seeds (Fig. 4A). Using 5 mM KNO_3_ also only caused partial dormancy release (G_MAX_ ~40%) in FH C24 seeds (Fig. 3A). This dormancy release by nitrate treatment in about half of the population’s seeds demonstrates that nitrate is indeed a major chemical involved in the GPAW-mediated seed dormancy release. However, other pathways must be involved in addition as for Air-GPAW full dormancy release of the seed population was achieved for C24 (Fig. 3A) and Col-0 (Fig. 4A). The control of germination by physiological dormancy includes the *DELAY OF GERMINATION 1* (*DOG1*) gene, which encodes a dormancy-specific master regulator (Graeber *et al.*, 2012; Nakabayashi *et al.*, 2012; Nee *et al.*, 2017; Nonogaki, 2017; Nishimura *et al.*, 2018). Due to the absence of dormancy, FH *dog1* mutant seeds fully germinated and no germination improvement was achieved by either GPAW or nitrate treatment (Fig. 4B, F). In agreement with GPAW not acting via altering the expression of *DOG1* in imbibed FH seeds, no differences in the *DOG1* transcript abundances were observed in response to GPAW or nitrate (Supplementary Fig. S3). Dormancy can also be released by AR storage (Fig. 1B) and, consequently, AR wild-type seed populations fully germinated (Fig. 4F). In contrast to FH Col-0 seeds and as for non-dormant *dog1* seeds, treatment of AR Col-0 seeds with either GPAW or nitrate did not appreciably affect the G_MAX_ responses of AR seeds.

Nitrate signalling in seeds to release physiological dormancy and promote germination is achieved via the NIN-LIKE PROTEIN 8 (NLP8) transcription factor which binds to the nitrate-responsive *cis*-element (NTR) in the promoter region of nitrate-responsive genes including *CYP707A2* (Nambara *et al.*, 2010; Nonogaki, 2017; Duermeyer *et al.*, 2018). In agreement with this, FH seeds of the *nlp8* mutant were dormant and did not respond to nitrate treatment with an increase in G_MAX_ because they were nitrate insensitive (Fig. 4C). In contrast to their insensitivity to nitrate, the FH *nlp8* seeds responded to Air-GPAW treatment with dormancy release, resulting in a maximum increase in G_MAX_ of ~60% (Fig. 4C). This is direct evidence that the dormancy release by Air-GPAW is not only caused by nitrate signalling, but by additional mechanisms triggered by other chemical species (Supplementary Fig. S2). The dose response of the FH *nlp8* seeds differs from that of FH wild-type (Col-0) seeds in that the maximum response (G_MAX_ ~60%) was already achieved with 15 min discharge time (Fig. 4F). In contrast to the FH Col-0 seed dose response where G_MAX_ further increased from ~60% (15 min) to ~90% (45 min), increased discharge time lowered the dormancy release response of the *nlp8* mutant, suggesting that multiple positive and negative pathways are involved (Fig. 4F). The response of FH *cyp707a2* mutant seeds to the treatments (Fig. 4D) has verified that the induction of the *CYP707A2* gene expression (Fig. 3) and thereby ABA degradation is a major requirement for dormancy release. While nitrate was completely ineffective in releasing their dormancy, with Air-GPAW, a partial release of FH *cyp707a2* seed dormancy was achieved (G_MAX_ ~30%), suggesting that mechanisms independent of ABA degradation are also triggered by GPAW. After-ripening fully released the dormancy of *nlp8* and *cyp707a2* seeds, and the G_MAX_ values of AR seeds were therefore not appreciably affected by GPAW or nitrate (Fig. 4F). The dormancy-releasing activity of the Air-GPAW is therefore not exclusively achieved by nitrate signalling via the NLP8 pathway, but via multiple pathways involving the concerted action of multiple chemical species in the GPAW.

Other major pathways involved in the control of physiological dormancy include RNS signalling (Supplementary Fig. S2) via ·NO, which is known to be generated *in planta* (Nambara *et al.*, 2010; Nonogaki, 2017; Kolbert *et al.*, 2019) and in GPAW (Lukes *et al.*, 2014; Takamatsu *et al.*, 2014; Zhou *et al.*, 2020). Signalling of ·NO in seeds includes crosstalk with ABA to increase ABA degradation, to inhibit ABA biosynthesis, and to remove ABA sensitivity by triggering proteolysis of the transcription factor ABA INSENSITIVE 5 (ABI5) (Bethke *et al.*, 2007; Holman *et al.*, 2009; Albertos *et al.*, 2015). The removal of the ABA sensitivity by ·NO signalling leading to proteasome-dependent ABI5 degradation can be achieved either by *S*-nitrosylation (Albertos *et al.*, 2015) or via the N-end rule pathway (Holman *et al.*, 2009). ABI5 degradation via the N-end rule pathway requires the E3 ubiquitin ligase PROTEOLYSIS 6 (PRT6) component, and *prt6* mutant seeds of *A. thaliana* are therefore hypersensitive to ABA (Holman *et al.*, 2009). In agreement with this, we found that FH *prt6* seeds exhibited an altered sensitivity to nitrate, and dormancy release was only observed with the highest nitrate concentration used (Fig. 4E). This resulted in a very different nitrate dose response (G_MAX_) of the *prt6* compared with Col-0 FH seeds (Fig. 4F). In contrast to this, the dose response of FH *prt6* seeds to Air-GPAW treatment was very similar to that of Col-0 FH seeds (Fig. 4A, E). In addition, it appears that GPAW does not act on altering the expression of *ABI5* in imbibed FH seeds (Supplementary Fig. S3). Air-GPAW-triggered dormancy release and germination in FH *nlp8* and *cyp707a2* seeds differed considerably from that of FH Col-0 seeds, whereas FH *prt6* seeds displayed a wild-type-like dose response (Fig. 4F). The repression of germination through prevention of testa rupture in *A. thaliana* requires ABI5 for down-regulation of expansin (*EXPA*) gene expression (Barros-Galvao *et al.*, 2019). Removal of ABA sensitivity by ABI5 proteolysis together with the accumulation of bioactive GA due to the GPAW-mediated induction of *GA3OX1* (Fig. 3) and reduced expression of *GA2OX2* (Supplementary Fig. S3) will therefore trigger CWRP gene expression to stimulate endosperm weakening and germination.

### Micropylar endosperm weakening is caused directly by GPAW-generated ROS as well as indirectly by GPAW-induced expression of cell wall-remodelling genes

**Fig. 5.**
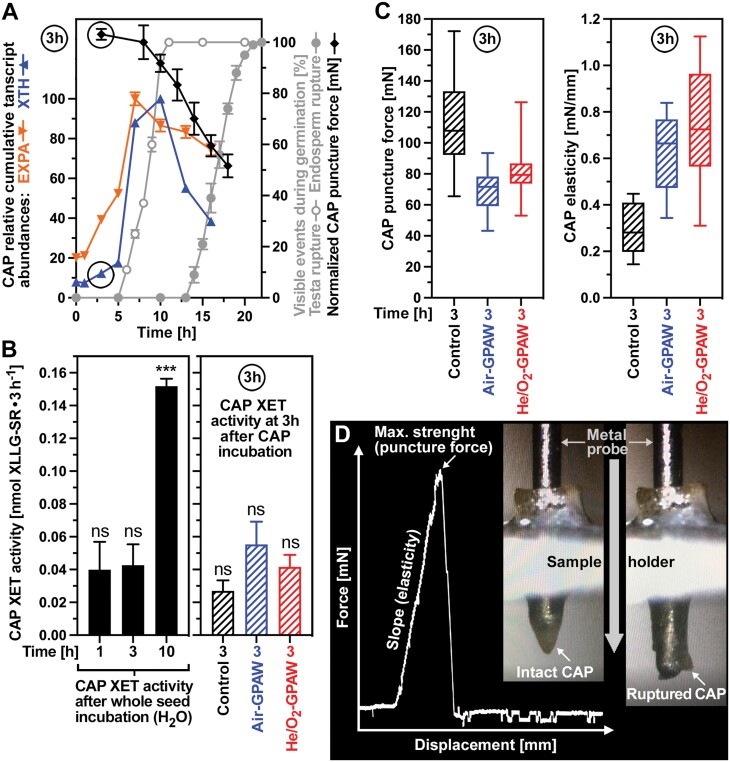
Biomechanical and biochemical analysis of GPAW-induced endosperm weakening. (A) Time courses of micropylar endosperm (cap) puncture force, testa and endosperm rupture, and expansin (*EXPA*) and xyloglucan endotransglycolases/hydrolase (*XTH*) transcript abundances in the cap of *Lepidium sativum* FR14 seeds during germination. For the cap puncture force, normalized values combined from two datasets (Linkies *et al.*, 2009; Graeber *et al.*, 2014) are presented. The cap-specific relative cumulative expression values of *EXPA* and *XTH* genes were from the spatiotemporal transcriptome dataset of *L. sativum* seed germination (Scheler *et al.*, 2015); for individual genes and details, see Supplementary Fig. S4. (B) Xyloglucan endotransglycosylase (XET) enzyme activities of XTH proteins in the cap after incubation of whole *L. sativum* seeds in dH_2_O for the times indicated (left panel). Effects of incubating isolated caps in Air-GPAW (45 min) or He/O_2_-GPAW (45 min) on the XET enzyme activities at 3 h (right panel). Note that only the 10 h CAP XET activity was statistically different, while all of the other XET activity values were not significantly (‘ns’) different from each other. (C) Biomechanical analysis of the effects of Air-GPAW (45 min) or He/O_2_-GPAW (45 min) on cap endosperm weakening at 3 h. The cap puncture force (tissue resistance) at 3 h (left panel) was determined as the maximal force from the displacement–force curve, and the cap tissue elasticity at 3 h (right panel) was calculated as the slope of the linear portion of the displacement–force curve. (D) Example displacement–force curve and images of the biomechancial assay with cap prior to rupture (intact) and cap post-rupture by the metal probe of the biomechanics device.

Endosperm weakening by cell wall loosening of the micropylar endosperm (cap) tissue precedes the completion of germination of non-dormant seeds by radicle emergence (Steinbrecher and Leubner-Metzger, 2017). The endosperm (aleurone layer) contributes to the coat-imposed dormancy of *A. thaliana* seeds (Fig. 1A), and the dormancy release, for example by ·NO and GA treatment, leads to *A. thaliana* cap weakening (Bethke *et al.*, 2007). Testa rupture is known to be preceded by the induction of cell wall-remodelling genes in the endosperm of *L. sativum* (Scheler *et al.*, 2015) and *A. thaliana* (Dekkers *et al.*, 2013) to promote endosperm weakening and testa rupture (Steinbrecher and Leubner-Metzger, 2017). Due to their larger size, the seeds of the Arabidopsis close relative *L. sativum* are highly suited for the direct biomechanical measurement of cap weakening by the puncture force method (Müller *et al.*, 2009; Graeber *et al.*, 2014; Steinbrecher and Leubner-Metzger, 2017). Figure 5A shows that the *L. sativum* cap puncture force was >100 mN in GPAW seeds imbibed for 3–8 h and subsequently decreased to ~92 mN (10 h) and 70 mN (14 h) in association with the progression with testa rupture. This was associated with a rapid rise of the *EXPA* and *XTH* transcript abundances in the cap. Transcript accumulation of all *EXPA* and *XTH* genes in the cap was 5-fold (at 7 h) and 8-fold (at 10 h), respectively (Fig. 5A). Transcript accumulation of *EXPA2* in the *L. sativum* cap was >60-fold (Supplementary Fig. S4) and *EXPA2* is also induced exclusively in the endosperm in germinating *A. thaliana* seeds (Supplementary Fig. S5). Most LesaXTH genes are mainly cap expressed, and the cumulative transcript abundance of all LesaXTH genes was several-fold higher in the cap as compared with the other seed compartments (Supplementary Fig S4E, F). We found that the accumulation of *XTH* transcripts in the *L. sativum* cap (Fig. 5A) was accompanied by a 4-fold increase in XET (EC 2.4.1.207) enzyme activity in the cap between 3 h and 10 h (Fig. 5B). The increased cap XET activity at 10 h is consistent with a role for XTHs in the subsequent cap weakening and testa rupture (Fig. 3A). In contrast to the enhanced cap XET activity between 3 h and 10 h, there was no difference in the cap XET activity between the 1 h and 3 h time points (Fig. 5B). Consistent with this, the onset of cap weakening was evident between 8 h and 10 h, and no decrease in cap puncture force was evident until 8 h (Fig. 5A).

In addition to ROS signalling and CWRPs (Supplementary Fig. S2), apoplastic ROS (aROS) produced in the plant cell wall can also act directly by chemical scission of backbone polysaccharides, resulting in cell wall loosening to enhance embryo elongation growth (Müller *et al.*, 2009; Bailly, 2019) and endosperm weakening (Müller *et al.*, 2009; Zhang *et al.*, 2014; Steinbrecher and Leubner-Metzger, 2017). Experimentally produced ·OH, for example, caused an ~50% decrease in the *L. sativum* cap puncture force within 1 h (Müller *et al.*, 2009). To test if the ·OH and other ROS/RNS produced in Air-GPAW and He/O_2_-GPAW (Fig. 2) cause cap weakening, we incubated isolated *L. sativum* caps in GPAW and biomechanically analysed their responses (Fig. 5). Figure 5C shows that the puncture force of caps incubated for 3 h in water (control) remained high (113.1 ± 4.9 mN) and therefore no cap weakening had occurred at the 3 h time point. In contrast to this, incubation of isolated caps in GPAW resulted in a significant decrease in the cap puncture force at the 3 h time point, with 70.5 ± 3.6 mN for Air-GPAW and 80.9 ± 2.3 mN for He/O_2_-GPAW (Fig. 5C). The XET enzyme activities of these GPAW-treated caps did not, however, differ from the control at 3 h (Fig. 5B). We therefore conclude that GPAW can cause cap weakening very early in imbibed seeds by direct chemical action of the ROS produced in the GPAW. The observed premature cap weakening caused by direct chemical action of the GPAW was associated not only with a decreased cap puncture force, but also with an increased cap elasticity compared with the control (Fig. 5C). Biological materials are structurally complex composites, and a decrease in puncture force has been observed in many endospermic seeds (Steinbrecher and Leubner-Metzger, 2017), but a change in the slope of the strain–stress curves (Fig. 5D; i.e. the cap elasticity) has not been previously reported for seeds. Whether the increase in the cap elasticity caused by reactive species in the GPAW (direct chemical scission of cell wall polysaccharides) is specific for the GPAW or also occurs in seeds upon abiotic stress is not known. Taken together, the GPAW-induced dormancy release is caused by multiple biochemical and biomechanical mechanisms which include the induction of endosperm weakening and the control of radicle emergence by ROS/RNS-mediated signalling and direct action.

## Discussion

### GPAW is an emerging seed treatment agri-technology to release physiological dormancy and stimulate germination by multiple molecular signalling pathways

Most crop species and their wild relatives produce seeds which are either non-dormant or physiologically dormant in their mature state at harvest (Finch-Savage and Leubner-Metzger, 2006; Nambara *et al.*, 2010; Holloway *et al.*, 2021). Examples for the latter include Brassicaceae crops such as oilseed rape, the *Brassica* vegetables, and *L. sativum* sprouts, and *A. thaliana* as their wild relative. We demonstrate here using dormant seeds of *A. thaliana* that GPAW treatment releases the physiological dormancy by triggering multiple key signalling pathways which gear the hormonal control towards the germination programme and by direct chemical action resulting in premature endosperm weakening. Depending on the carrier gas (air or He/O_2_) and the discharge treatment time (bubble reactor; Supplementary Fig. S1), the GPAW used to imbibe the seeds differed qualitatively and quantitatively in the cocktail of reactive chemical species they contain (secondary reactions in Fig. 2). Major chemical species quantified included NO_3_^–^ (only in Air-GPAW), and H_2_O_2_ and the ·OH radical (10-fold higher concentration in He/O_2_-GPAW compared with Air-GPAW). These, and in addition the ·NO generated from NO_3_^–^ and H_2_O_2_, are known to be produced by seeds in response to environmental cues to release physiological dormancy via well-established molecular signalling pathways (Supplementary Fig. S2). We demonstrate here by using specific *A. thaliana* mutants that GPAW triggers many of these pathways which interact synergistically to cause the observed strong molecular and physiological responses. These include the NLP8-mediated nitrate signalling pathway that induces *CYP707A2* gene expression as a key target (Kushiro *et al.*, 2004; Okamoto *et al.*, 2006; Matakiadis *et al.*, 2009; Yan *et al.*, 2016; Duermeyer *et al.*, 2018). Non-NLP8 RNS (·NO) signalling pathways to remove ABA sensitivity by ABI5 degradation are also involved (Bethke *et al.*, 2007; Holman *et al.*, 2009; Albertos *et al.*, 2015; Nonogaki, 2017; Kolbert *et al.*, 2019), as well as ROS signalling pathways that up-regulate GA biosynthesis (*GA3OX1*) and CWRP genes from the EXPA and XTH families (Chen *et al.*, 2002; Voegele *et al.*, 2011; Graeber *et al.*, 2014; Steinbrecher and Leubner-Metzger, 2017, 2022; Barros-Galvao *et al.*, 2019; Sanchez-Montesino *et al.*, 2019; Herburger *et al.*, 2020).

### GPAW-generated ROS cause micropylar endosperm (cap) weakening by direct chemical action and by inducing *EXPA* and *XTH* gene expression

Direct biomechanical quantification of the cap puncture force conducted in *L. sativum* seeds demonstrated that GPAW causes premature endosperm weakening that is detectable just 3 h after imbibition with GPAW (Fig. 5). Endosperm weakening is a prerequisite for the completion of germination by radicle protrusion and is blocked in physiologically dormant seeds as a component of the coat-imposed dormancy mechanism (Bethke *et al.*, 2007; Graeber *et al.*, 2014; Steinbrecher and Leubner-Metzger, 2017). In particular, endosperm weakening is inhibited by ABA, and promoted by GA and aROS (Bethke *et al.*, 2007; Müller *et al.*, 2009; Graeber *et al.*, 2014; Yan *et al.*, 2014; Zhang *et al.*, 2014; Steinbrecher and Leubner-Metzger, 2017; Bailly, 2019). The aROS, including ·OH and O_2_·^–^, are produced *in vivo* in the cap cell walls of *L. sativum* and lettuce seeds, and direct ·OH attack of cell wall polysaccharides has been demonstrated to cause *in vivo* polysaccharide scission and therefore constitutes a more direct mechanism of cap weakening (Müller *et al.*, 2009; Zhang *et al.*, 2014; Steinbrecher and Leubner-Metzger, 2017). It is also not known if GPAW treatment of seeds causes a similar weakening of the non-micropylar endosperm. It is, however, known that micropylar (cap) and non-micropylar endosperm of *L. sativum* differ in the degree of the weakening in that it is only pronounced in the cap and comparatively small in other regions of the endosperm (Lee *et al.*, 2012). Similar findings were made in endospermic seeds of other species (Steinbrecher and Leubner-Metzger, 2017). In addition, seeds contain seed coats and in many cases fruit coats which may differ locally in their permeability for compound uptake (Hermann *et al.*, 2007; Scheler *et al.*, 2015; Steinbrecher and Leubner-Metzger, 2017); this could also differ for GPAW.

We found that the GPAW-induced premature cap weakening at 3 h was not associated with the induction of XET enzyme activity (Fig. 5), but by the direct chemical action of ·OH radicals in Air-GPAW and He/O_2_-GPAW (Fig. 2). Further progression of cap weakening at ~10 h and later, however, involves the GPAW-enhanced up-regulation of XTH genes and accumulation of XET enzyme activity in the cap (Fig. 5). Consistent with this, the GA-regulated *LeXET4* gene is induced during tomato cap weakening (Chen *et al.*, 2002) and the *XTH18* and *XTH19* genes are expressed in *A. thaliana* and *L. sativum* in a GA-regulated manner (Voegele *et al.*, 2011; Graeber *et al.*, 2014). XTHs with XET enzyme activity can, however, also reinforce tissues and play roles in the coleorhiza-enforced dormancy in grasses, which is—together with the endosperm-imposed dormancy of eudicot seeds—an example for the convergent evolution of mechanical restraint by overlaying tissues (Yan *et al.*, 2014; Holloway *et al.*, 2021). Interestingly, and in agreement with a role in promoting endosperm weakening and testa rupture, most of the *XTH* genes are expressed in the endosperm upon GPAW treatment. About half of the *XTH* genes are differentially expressed in that they are up-regulated upon testa rupture in *L. sativum* (Supplementary Fig. S4) and *A. thaliana* (Supplementary Fig. S5). GPAW therefore acts by mimicking environmental cues which trigger the removal of the various layers of dormancy blocks to permit seed germination.

### Practical applications

Innovations in seed treatment technologies support primary crop production and increasing yield potential by providing protection against pathogens and physiological enhancement to perform better in abiotic stress conditions. We demonstrated that the hallmark of GPAW action important for developing seed treatment and plant growth applications is the concerted action of the combined major chemical species produced to trigger multiple molecular direct (chemical weakening) and indirect (signalling pathways) mechanisms. GPAW thereby mimicks the multitude of environmental signals which are sensed and integrated by seeds. Among them are signalling pathways which are conserved among plants (Supplementary Fig. S2) and allow translation of our findings to seeds of other species for practical applications. The knowledge of the underpinning mechanisms derived from our work is therefore crucial for the emerging ‘plasma agriculture’ (Bourke *et al.*, 2018; Ito *et al.*, 2018; Ranieri *et al.*, 2021) in which non-thermal atmospheric gas plasma is developed into environment-friendly agri-technologies for the sustainable global food production.

## Supplementary data

The following supplementary data are available at *JXB* online.

Fig. S1. Diagram of the bubble reactor used to produce GPAW.

Fig. S2. Schematic presentation of ROS and RNS signalling pathways.

Fig. S3. GPAW-induced gene expression in seeds.

Fig. S4. Cell wall-remodelling genes in germinating *L. sativum* seeds.

Fig. S5. Cell wall-remodelling genes in germinating *A. thaliana* seeds.

Table S1. Primer sequences used for RT–qPCR.

erac150_suppl_Supplementary_figures_S1-S5_Supplementary_table_S1

## Data Availability

All data presented or analysed in this article are available online through figshare https://doi.org/10.17637/rh.19376306 and in the supplementary data.
