# Supplementary material for: Molecular mechanisms of seed dormancy release by gas plasma-activated water technology
Source: J Exp Bot. 2022 Apr 15;73(12):4065–78. doi: 10.1093/jxb/erac150 (PMC9232203; doi:10.1093/jxb/erac150)
Supplement: erac150_suppl_Supplementary_figures_S1-S5_Supplementary_table_S1 [file erac150_suppl_supplementary_figures_s1-s5_supplementary_table_s1.pdf]

Molecular mechanisms of seed dormancy release by gas plasma-activated water technology

Giles Grainge[1], Kazumi Nakabayashi[1], Tina Steinbrecher[1], Sue Kennedy[2], Junchen Ren[3], Felipe Iza[3,4] and Gerhard Leubner-Metzger[1,5,*]

[1] Department of Biological Sciences, Royal Holloway University of London, Egham, Surrey, TW20 0EX, United Kingdom, Web: 'The Seed Biology Place' - www.seedbiology.eu
[2] Elsoms Seeds Ltd, Spalding, Lincolnshire, PE11 1QG, United Kingdom
[3] Wolfson School of Mechanical, Electrical and Manufacturing Engineering, Loughborough University, Leicestershire, LE11 3TU, United Kingdom
[4] Division of Advanced Nuclear Engineering, Pohang University of Science and Technology (POSTECH), Pohang, Gyeongbuk 790-784, South Korea
[5] Laboratory of Growth Regulators, Institute of Experimental Botany, Czech Academy of Sciences and Faculty of Science, Palacký University Olomouc, CZ-78371 Olomouc, Czech Republic

[*] Correspondence: Gerhard Leubner-Metzger (gerhard.leubner@rhul.ac.uk)
Telephone number: (0)+44 1784 443553
URL: www.seedbiology.eu and https://pure.royalholloway.ac.uk/portal/en/persons/gerhard-leubner(b07cd3da-9c1d-4167-8d52-199a13d54351).html

Journal of Experimental Botany: Research Article

Supplementary data

© The Author(s) 2022. Published by Oxford University Press on behalf of the Society for Experimental Biology.
This is an Open Access article distributed under the terms of the Creative Commons Attribution License (https://creativecommons.org/licenses/by/4.0/), which permits unrestricted reuse, distribution, and reproduction in any medium, provided the original work is properly cited.

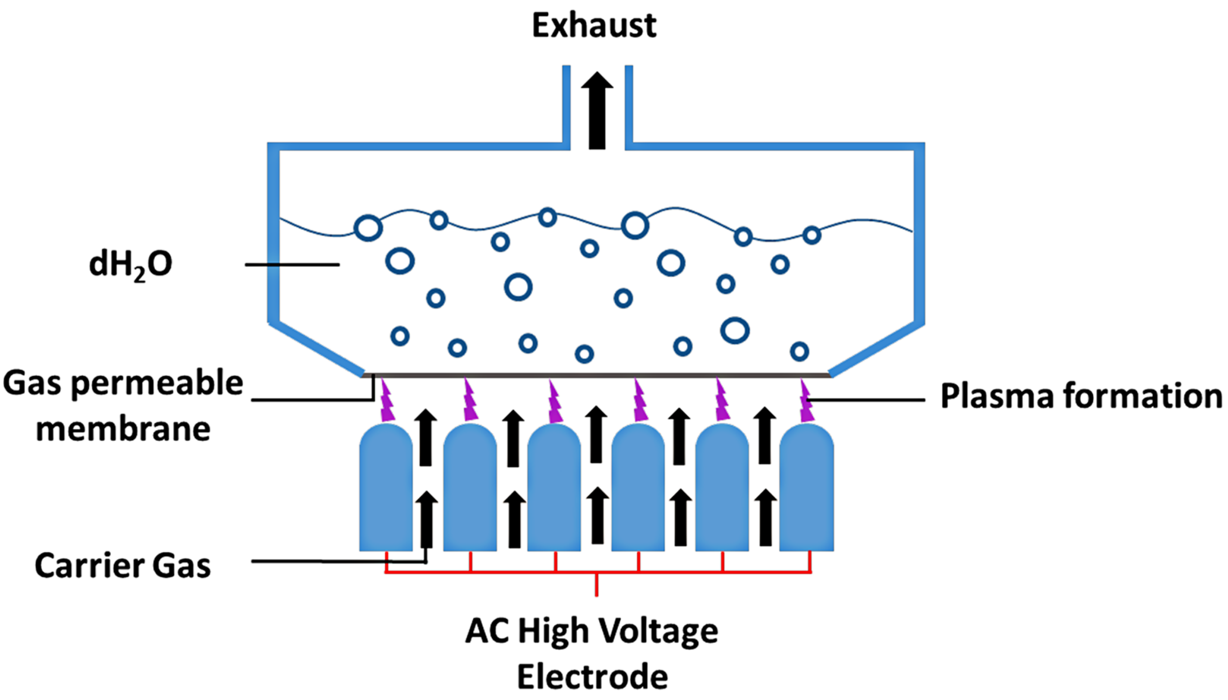

Supplementary Fig. S1 Diagram of the bubble reactor used to produce gas plasma-activated water (GPAW). The bubble reactor includes 12 high voltage AC electrodes in a dielectric material fixed below a gas permeable stainless-steel membrane. Above the membrane is a tank containing 100 ml of deionised (dH_2O; water purifier system Select Purewater 300, Purite Ltd., Trevose, Pennsylvania, USA). Carrier gas flows past the electrode, and then through the membrane and dH_2O. For activation, plasma is formed between the electrodes and the membrane within the carrier gas and then flows through the membrane bubbling up through the water to produce the GPAW. Major chemical species produced with the bubble reactor were quantified (Figure 2). The non-equilibrium chemistry triggered by atmospheric pressure plasmas in contact with water is complex (Bruggeman *et al.*, 2016; Lu *et al.*, 2016) and here we highlight only some of the key pathways that lead to the formation of the reactive species that have been identified to play a concerted role in the release of physiological dormancy of seeds, namely NO_3^-, $^{\bullet}OH$, H_2O_2 and $^{\bullet}NO$.

$^{\bullet}$OH (hydroxyl radical):

Although reactive plasma species such as O, $^1O_2$, $^{\bullet}H$ and $HO_2^{\bullet}$ as well as VUV radiation can dissociate water molecules and produce hydroxyl radicals (Bruggeman *et al.*, 2016), hydroxyl radicals in plasma systems are primarily formed at the gas liquid interface by electron impact dissociation [1] of water molecules (Vasko *et al.*, 2014):

$$e + H_2O \rightarrow e + {}^{\bullet}OH + {}^{\bullet}H \qquad [1]$$

$^{\bullet}$OH radicals are short-lived and therefore they do not contribute to the $^{\bullet}$OH radicals observed hours after the plasma treatment. Instead, in GPAW, $^{\bullet}$OH radicals keep being produced well after the plasma treatment has ended via secondary reactions such as Fenton reactions when metal ions are present [2], quenching of hydrogen peroxide by long lived species such as ozone [3] and decomposition of peroxynitrite [4], which forms in the water as a result of reactions of reactive oxygen and nitrogen species species (Bruggeman *et al.*, 2016; Lukes *et al.*, 2014):

$$Fe^{2+} + 2H_2O_2 \rightarrow Fe^{3+} + {}^{\bullet}OH + HO_2^{\bullet} + H_2O \quad [2]$$

$$O_3 + H_2O_2 \rightarrow HO_2^{\bullet} + {}^{\bullet}OH + O_2 \quad [3]$$

$$O{=}NOOH \rightarrow {}^{\bullet}NO_2 + {}^{\bullet}OH \quad [4]$$

H_2O_2 (hydrogen peroxide):

The main reaction leading to the formation of H_2O_2 is the recombination of hydroxyl radicals [5] (Vasko *et al.*, 2014; Winter *et al.*, 2014):

$$ {}^{\bullet}OH + {}^{\bullet}OH \rightarrow H_2O_2 \quad [5]$$

Unlike hydroxyl radicals, hydrogen peroxide is fairly long-lived and can be detected in GPAW long after the plasma treatment has ended. Reactions contributing to the decay over time of H_2O_2 include the ozone and iron catalysed decomposition reactions (2 and 3) and in acidic conditions, the reaction with nitrite ions to form peroxynitrite [6] (Lukes *et al.*, 2014):

$$NO_2^- + H_2O_2 + H^+ \rightarrow O{=}NOOH + H_2O \quad [6]$$

NO_2^- (nitrite) and NO_3^- (nitrate):

Nitrites and nitrates are formed in plasma-treated water through dissolution of nitrogen oxides, nitrous acid and nitric acid formed in the plasma by gas-phase reactions of dissociated N_2, O_2 and H_2O [7-10] (Bruggeman *et al.*, 2016; Lukes *et al.*, 2014; Sakiyama *et al.*, 2012):

$$HNO_3 \rightarrow NO_3^- + H^+ \quad [7]$$

$$HNO_2 \rightarrow NO_2^- + H^+ \quad [8]$$

$$ {}^{\bullet}NO_2 + {}^{\bullet}NO_2 + H_2O \rightarrow NO_2^- + NO_3^- + 2H^+ \quad [9]$$

$$ {}^{\bullet}NO + {}^{\bullet}NO_2 + H_2O \rightarrow 2NO_2^- + 2H^+ \quad [10]$$

The relative concentrations of NO_3^-, NO_2^- and H_2O_2 in GPAW under acidic conditions is regulated by peroxynitrite, which favours the formation of nitrate over nitrite and hydrogen peroxide over time [4,6] (Lukes *et al.*, 2014).

${}^{\bullet}NO$ (nitric oxide):

Nitric oxide is produced in the gas plasma as a result of the dissociation of N_2 and O_2 and can partly dissolve in water before it is converted into other NO_x species [11-13] (Sakiyama *et al.*, 2012):

$$N_2^* + O \rightarrow {}^{\bullet}NO + {}^{\bullet}N \quad [11]$$

$$O_2 + {}^{\bullet}N \rightarrow {}^{\bullet}NO + O \quad [12]$$

$$ {}^{\bullet}N + {}^{\bullet}OH \rightarrow {}^{\bullet}NO + {}^{\bullet}H \quad [13]$$

Besides direct solvation, ${}^{\bullet}NO$ is also produced at the liquid interface and inside water by reduction of plasma generated nitrogen dioxide [14,15] (Jablonowski *et al.*, 2018):

$$ {}^{\bullet}NO_2 + O \rightarrow {}^{\bullet}NO + O_2 \quad [14]$$

$$ {}^{\bullet}NO_2 + O_3 \rightarrow {}^{\bullet}NO + 2O_2 \quad [15]$$

Seed dormancy releasing pathways of major chemical species in GPAW

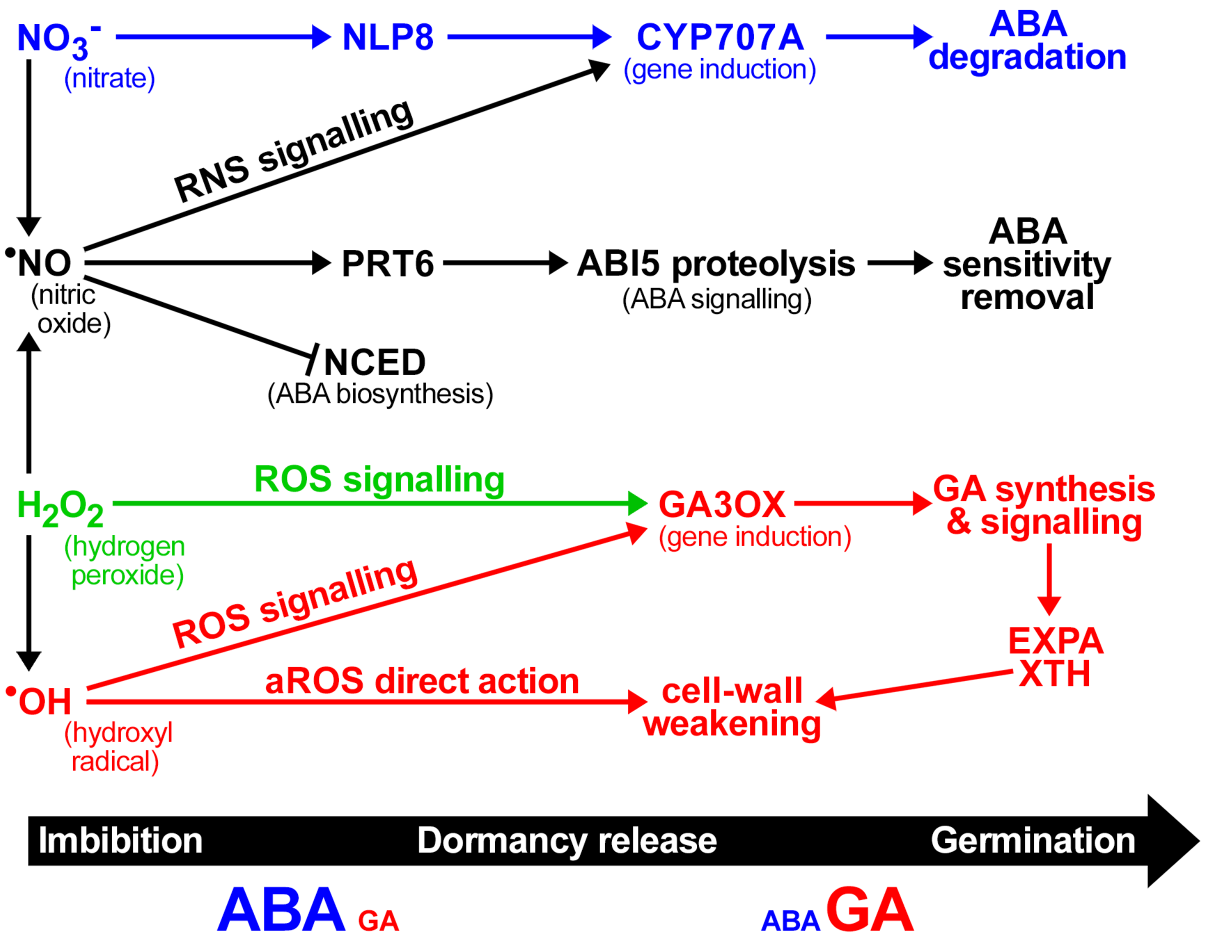

Supplementary Fig. S2 Simplified schematic presentation of ROS and RNS signalling pathways in plants. Major chemical species produced in GPAW include NO_3^-, $^{\bullet}NO$, H_2O_2 and $^{\bullet}OH$ (Figure 2, Supplementary Figure S1) which are also produced *in planta* and are known for their signalling roles (Nonogaki, 2017) and direct chemical actions on cell walls (Müller *et al.*, 2009). In brief, in imbibed seeds the *CYP707A2* gene encoding ABA 8'-hydroxylase to catalyse ABA degradation , is known to be induced by NO_3^- via the NLP8 master regulator (Duermeyer *et al.*, 2018; Nonogaki, 2017; Yan *et al.*, 2016). RNS signalling by $^{\bullet}NO$ which is known to be generated in planta (Kolbert *et al.*, 2019; Liu and Zhang, 2009) also leads to reduced ABA biosynthesis and by signalling via E3 ubiquitin ligase PRT6 (as depicted in the simplified scheme) and other components of the N-end rule pathway (Holdsworth *et al.*, 2020; Holman *et al.*, 2009) or by *S*-nitrosylation (not presented in the simplified scheme) (Albertos *et al.*, 2015) to the removal of ABA sensitivity by ABI5 proteolysis. ROS signalling by $^{\bullet}OH$, H_2O_2 and other ROS leads in seeds to the induction of the *GA3OX* genes to catalyse the biosynthesis of bioactive GA (Bailly, 2019; Liu *et al.*, 2010). High H_2O_2 concentrations (5-10 mM) are required for the very early up-regulation of *GA3OX1* and *CYP707A2* genes in imbibed seeds, low H_2O_2 concentrations (< 1mM) are less effective (Liu *et al.*, 2010). Apoplastic ROS (aROS) produced in the cell wall of seed compartments are involved in embryo expansion growth and micropylar endosperm weakening (Graeber *et al.*, 2014; Müller *et al.*, 2009; Steinbrecher and Leubner-Metzger, 2017; Zhang *et al.*, 2014). Experimentally produced $^{\bullet}OH$ (Fenton reaction) for example caused a ca. 50% decrease in the *L. sativum* CAP puncture force within one hour (Müller *et al.*, 2009). Expansins (EXPA) and xyloglucan endo-transglycosylases/hydrolases (XTH) including through their xyloglucan

endo-transglycosylase enzyme activity (XET) enzyme activity are involved in promoting testa rupture and enhanced endosperm CAP weakening (Chen *et al.*, 2002; Graeber *et al.*, 2014; Steinbrecher and Leubner-Metzger, 2017; Voegele *et al.*, 2011). Due to the altered balance in GA and ABA metabolism and sensitivity release dormancy and shift the seed state towards the germination programme (Finch-Savage and Leubner-Metzger, 2006).

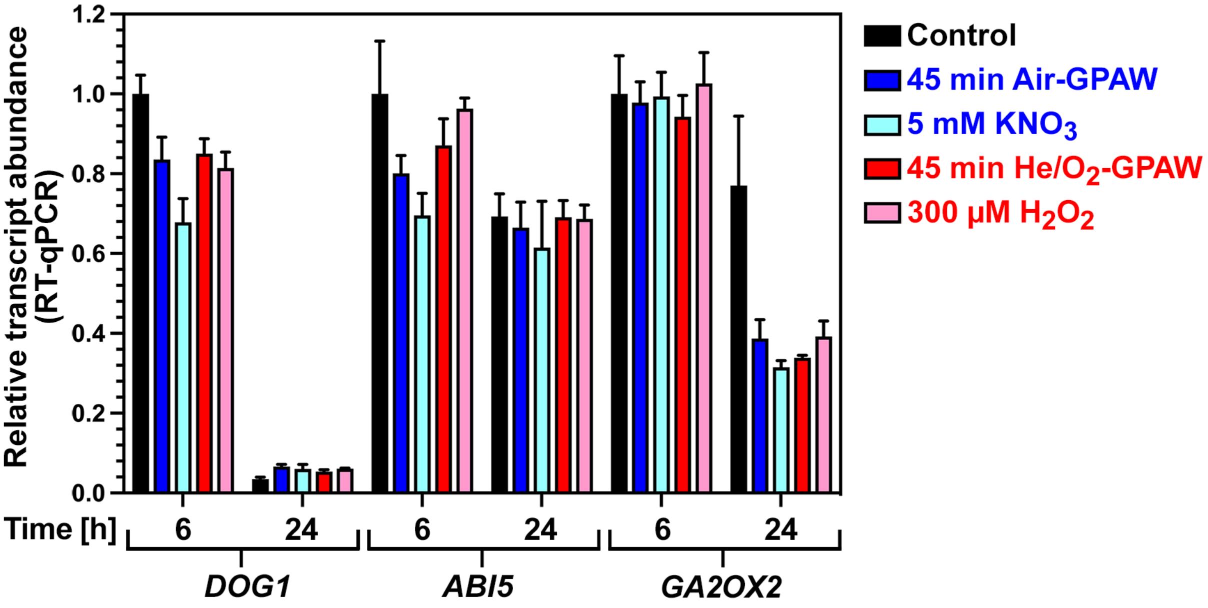

Supplementary Fig. 3 GPAW-induced gene expression associated with dormancy release and germination. RT-qPCR analyses of *Arabidopsis thaliana* C24 seed transcript abundances at 6 h and 24 h, as indicated, for key genes encoding the dormancy master regulator (*DOG1*), a transcription factor conferring seed ABA sensitivity (*ABI5*), and a GA inactivation enzyme (*GA2OX2*) known to be involved in dormancy and germination. Relative mean ± SEM values compared to the 6-h control (set to 1 for each gene) are presented for the control, Air-GPAW, He/O_2-GPAW, NO_3^- and H_2O_2 treatments. Relative mean ± SEM values compared to the 6-h control are presented.

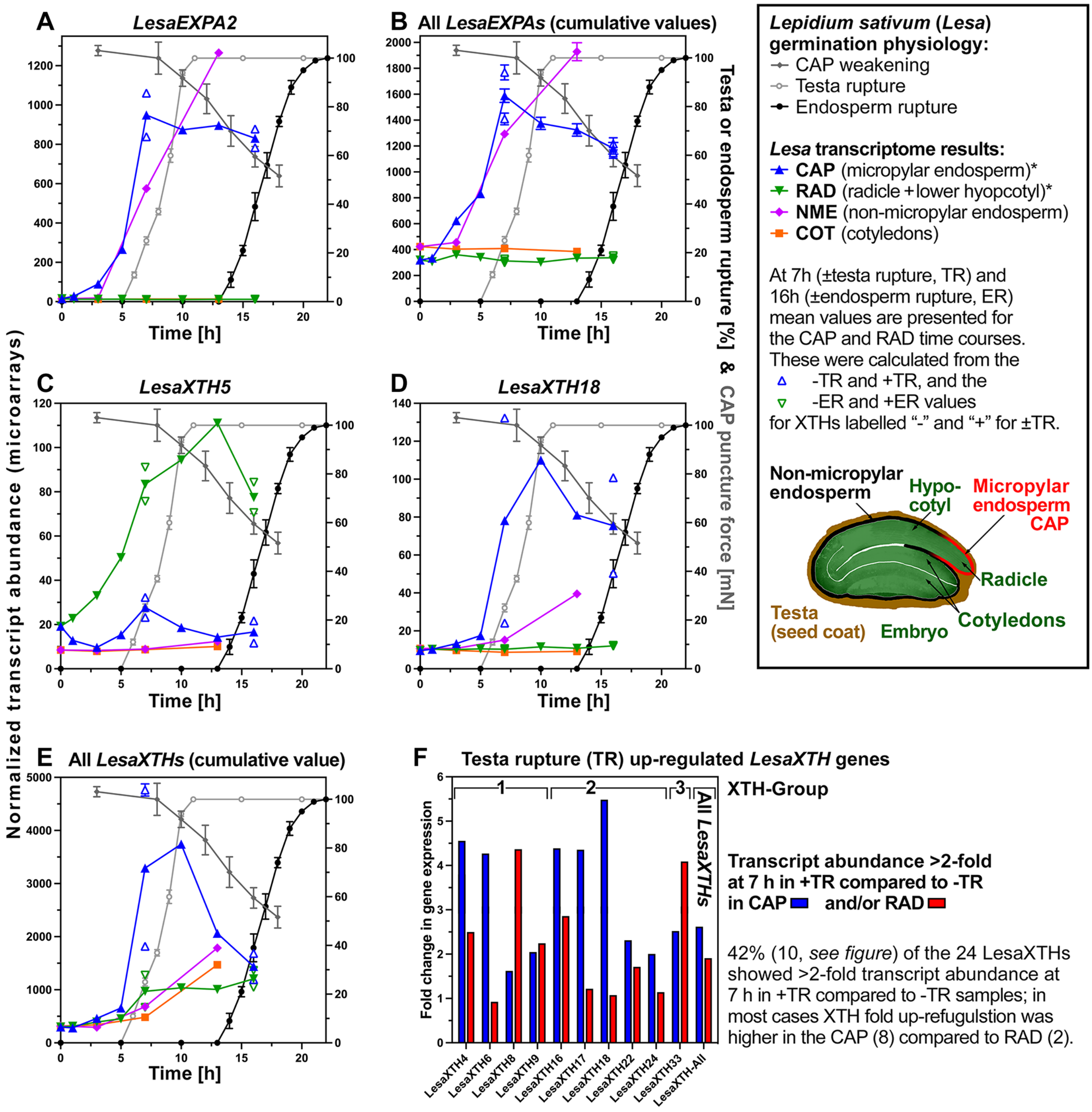

Supplementary Fig. S4 Spatiotemporal expression of cell wall remodelling genes in germinating *Lepidium sativum* seeds. Transcriptome analysis (microarrays) of *EXPA* and *XTH* gene expression in *L. sativum* FR14 seed compartments (Scheler *et al.*, 2015) as specified in the legend. (A) *LesaEXPA2*, for which the expression is endosperm-specific. (B) Cumulative values for all 18 detected *LesaEXPA* genes (*EXPA1,2,4,6,7,8,9,10,11,12,13,14,15,16,17,18,20,21*). (C) *LesaXTH5*. (D) *LesaXTH18*. (E) Cumulative values for all 24 detected *LesaXTH* genes (*XTH1,4,5,6,8,9,10,13, 15,16,17,18,19,20,22,23,24,25,27,28,30,31,32,33*). (F) Interestingly, and in agreement with a role of in promoting endosperm weakening and testa rupture, most of the *XTH* genes are expressed in the endosperm and about half of the *XTH* genes are differentially expressed in that they are, as upon GPAW treatment, up-regulated upon testa rupture in *L. sativum* and *A. thaliana* (Supplementary Figures S4F and S5).

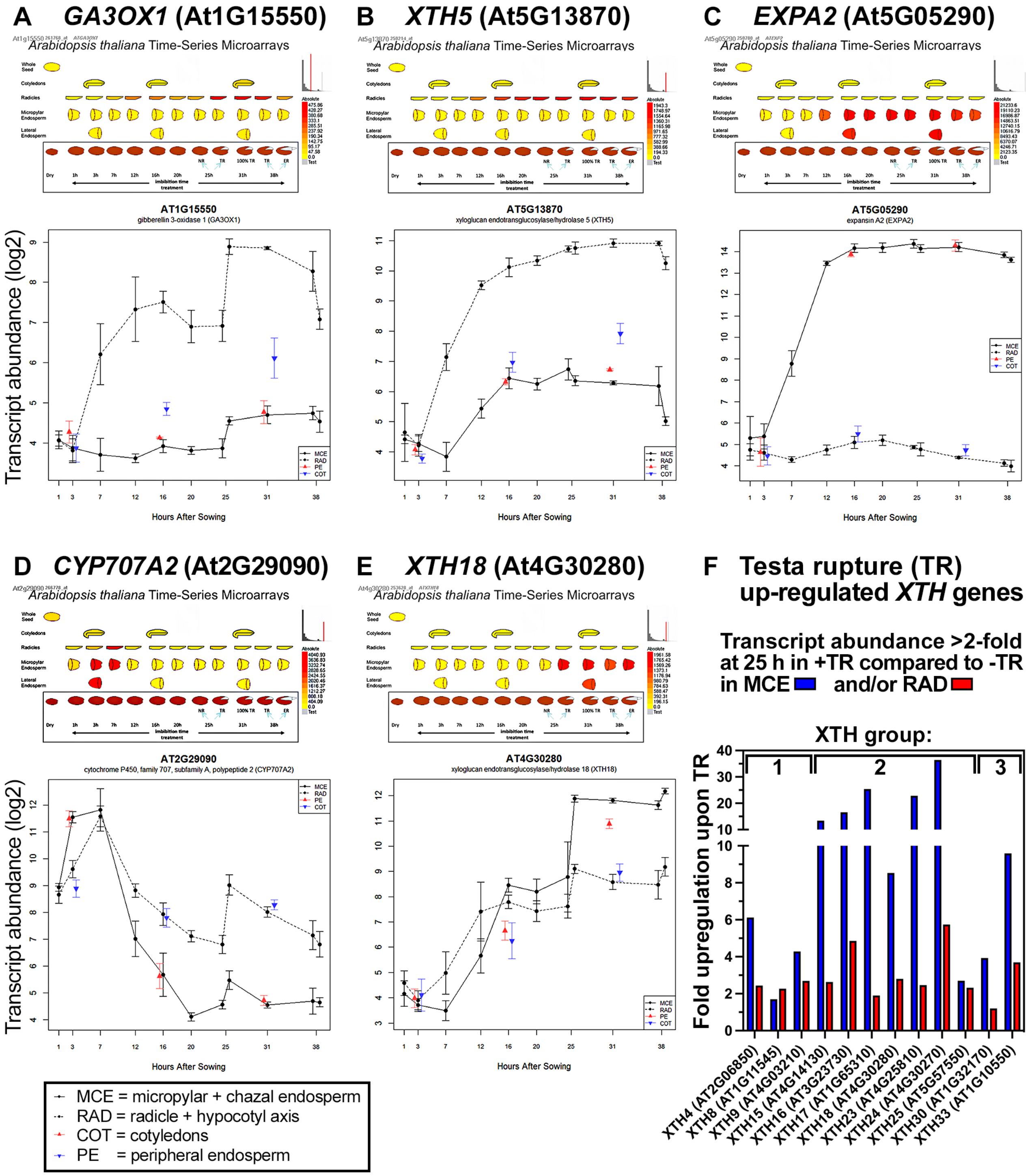

Supplementary Fig. S5 Spatiotemporal expression of cell wall remodelling genes in germinating *Arabidopsis thaliana* seeds. Transcriptome analysis (microarrays) of *EXPA, XTH*, and hormone-related gene expression in *A. thaliana* seed compartments (Dekkers *et al.*, 2013) as specified in the legend. (A) *AtGA3OX1*. (B) *AtXTH5*. (C) *AtEXPA2*. (D) *AtCYP707A2*. (E) *AtXTH18*. (F) *Arabidopsis thaliana XTH* genes up-regulated upon testa rupture; note that Dekkers et al. (2013) identified 503 genes in the endosperm and 283 genes in the radicle which are upregulated by testa rupture. Transcript abundances (log2) (Dekkers *et al.*, 2013) from the eFP browser (Winter *et al.*, 2007) are presented.

Supplementary Table S1 Primer sequences used for RT-qPCR

Gene name	Gene ID		Primer sequences (5’-3’)	Annealing Temp (°C)[b]	Refer-ence
CYP707A2	At2g29090	Fw	CGTCTCTCACATCGAGCTCCTT	60	[1]
		Rev	CCAAAAGTCCATCAACACCCTC		
GA3OX1	At1g15550	Fw	TCCGAAGGTTTCACCATCACT	60	[2]
		Rev	TCGCAGTAGTTGAGGTGATGTTG		
NCED2	At4g18350	Fw	GCGGCTGAGCGTGCATTAA	60	[3]
		Rev	GGGAATAATTCCCGGCAATCT		
NCED9	At1g78390	Fw	GGAAAACGCCATGATCTCACA	60	[3]
		Rev	AGGATCCGCCGTTTTAGGAT		
XTH5	At5g13870	Fw	CACGTCGATGGATGTGAAGCT	64	[4]
		Rev	CTTTCTGATCCCACCAACGTTT		
EXPA1	At1g69530	Fw	AACGCACACGCCACATTCTAC	64	[5]
		Rev	CGTGTTGGTTCCATAGCCTTG		
EXPA2	At5g05290	Fw	CATAAACTCCGACGACAACG	64	[6]
		Rev	TACCCACAAGCACCACCCAT		
EXPA8	At2g40610	Fw	GCTCAAAAACACAGTCGTGGC	64	[5]
		Rev	CGTTACCTGGAAGGAAAGGCT		
DOG1	At5g45830	Fw	GAGCTGATCTTGCTCACCGATGTAG	60	[7]
		Rev	CCGCCACCACCTGAAGATTCGTAG		
ABI5	At2g36270	Fw	CAGCTGCAGGTTCACATTCTG	60	[2]
		Rev	CACCCTCGCCTCCATTGTTAT		
GA2OX2	At1g30040	Fw	CCTAAAACCTCCGCCGTTTT	60	[2]
		Rev	CCTTCATGTACTCCTCCACCGA		
Hobbit[a]	At2g20000	Fw	ACAAGACACTACAACGCATGGTAC	60	[7]
		Rev	TCTCTAGTGCTTCCTCACTTCTCTTC		
TIP41-Like[a]	At4g34270	Fw	GTGAAAACTGTTGGAGAGAAGCAA	60	[8]
		Rev	TCAACTGGATACCCTTTCGCA		[9]

[a] Reference gene; [b] Annealing temperature used in qPCR assays; [c] References: [1] (Kushiro *et al.*, 2004), [2] (Ogawa *et al.*, 2003), [3] (Seo *et al.*, 2004), [4] (Liu *et al.*, 2010), [5] (Sanchez-Montesino *et al.*, 2019), [6] (Yan *et al.*, 2014), [7] (Nakabayashi *et al.*, 2012), [8] (Czechowski *et al.*, 2005), [9] This study.

References used in Supplementary figure legends and tables

Albertos P, Romero-Puertas MC, Tatematsu K, Mateos I, Sanchez-Vicente I, Nambara E, Lorenzo O. 2015. S-nitrosylation triggers ABI5 degradation to promote seed germination and seedling growth. Nature Communications **6**, 8669.

Bailly C. 2019. The signalling role of ROS in the regulation of seed germination and dormancy. Biochemical Journal **476**, 3019-3032.

Bruggeman PJ, Kushner MJ, Locke BR, Gardeniers JGE, Graham WG, Graves DB, Hofman-Caris RCHM, Maric D, Reid JP, Ceriani E, Rivas DF, Foster JE, Garrick SC, Gorbanev Y, Hamaguchi S, Iza F, Jablonowski H, Klimova E, Kolb J, Krcma F, Lukes P, Machala Z, Marinov I, Mariotti D, Thagard SM, Minakata D, Neyts EC, Pawlat J, Petrovic ZL, Pflieger R, Reuter S, Schram DC, Schroter S, Shiraiwa M, Tarabova B, Tsai PA, Verlet JRR, von Woedtke T, Wilson KR, Yasui K, Zvereva G. 2016. Plasma-liquid interactions: a review and roadmap. Plasma Sources Science & Technology **25**.

Chen F, Nonogaki H, Bradford KJ. 2002. A gibberellin-regulated xyloglucan endotransglycosylase gene is expressed in the endosperm cap during tomato seed germination. Journal of Experimental Botany **53**, 215-223.

Czechowski T, Stitt M, Altmann T, Udvardi MK, Scheible WR. 2005. Genome-wide identification and testing of superior reference genes for transcript normalization in Arabidopsis. Plant Physiol **139**, 5-17.

Dekkers BJW, Pearce S, van Bolderen-Veldkamp RPM, Marshall A, Wider P, Gilbert J, Drost H-G, Bassel GW, Müller K, King JR, Wood ATA, Grosse I, Quint M, Krasnogor N, Leubner-Metzger G, Holdsworth MJ, Bentsink L. 2013. Transcriptional dynamics of two seed compartments with opposing roles in Arabidopsis seed germination. Plant Physiology **163**, 205-215.

Duermeyer L, Khodapanahi E, Yan DW, Krapp A, Rothstein SJ, Nambara E. 2018. Regulation of seed dormancy and germination by nitrate. Seed Science Research **28**, 150-157.

Finch-Savage WE, Leubner-Metzger G. 2006. Seed dormancy and the control of germination. New Phytologist **171**, 501-523.

Graeber K, Linkies A, Steinbrecher T, Mummenhoff K, Tarkowská D, Turečková V, Ignatz M, Sperber K, Voegele A, de Jong H, Urbanová T, Strnad M, Leubner-Metzger G. 2014. *DELAY OF GERMINATION 1* mediates a conserved coat dormancy mechanism for the temperature- and gibberellin-dependent control of seed germination. PNAS **111**, E3571-E3580.

Holdsworth MJ, Vicente J, Sharma G, Abbas M, Zubrycka A. 2020. The plant N-degron pathways of ubiquitin-mediated proteolysis. Journal of Integrative Plant Biology **62**, 70-89.

Holman TJ, Jones PD, Russell L, Medhurst A, Ubeda Tomas S, Talloji P, Marquez J, Schmuths H, Tung SA, Taylor I, Footitt S, Bachmair A, Theodoulou FL, Holdsworth MJ. 2009. The N-end rule pathway promotes seed germination and establishment through removal of ABA sensitivity in Arabidopsis. Proc Natl Acad Sci U S A **106**, 4549-4554.

Jablonowski H, Schmidt-Bleker A, Weltmann KD, von Woedtke T, Wende K. 2018. Non-touching plasma-liquid interaction - where is aqueous nitric oxide generated? Physical Chemistry Chemical Physics **20**, 25387-25398.

Kolbert Z, Barroso JB, Brouquisse R, Corpas FJ, Gupta KJ, Lindermayr C, Loake GJ, Palma JM, Petrivalsky M, Wendehenne D, Hancock JT. 2019. A forty year journey: The generation and roles of NO in plants. Nitric Oxide-Biology and Chemistry **93**, 53-70.

Kushiro T, Okamoto M, Nakabayashi K, Yamagishi K, Kitamura S, Asami T, Hirai N, Koshiba T, Kamiya Y, Nambara E. 2004. The *Arabidopsis* cytochrome P450 CYP707A encodes ABA 8'-hydroxylases: key enzymes in ABA catabolism. European Molecular Biology Organization Journal **23**, 1647-1656.

Liu Y, Ye N, Liu R, Chen M, Zhang J. 2010. H_2O_2 mediates the regulation of ABA catabolism and GA biosynthesis in *Arabidopsis* seed dormancy and germination. Journal of Experimental Botany **61**, 2979-2990.

Liu Y, Zhang J. 2009. Rapid accumulation of NO regulates ABA catabolism and seed dormancy during imbibition in Arabidopsis. Plant Signaling & Behavior **4**, 905-907.

Lu X, Naidis GV, Laroussi M, Reuter S, Graves DB, Ostrikov K. 2016. Reactive species in non-equilibrium atmospheric-pressure plasmas: Generation, transport, and biological effects. Physics Reports-Review Section of Physics Letters **630**, 1-84.

Lukes P, Dolezalova E, Sisrova I, Clupek M. 2014. Aqueous-phase chemistry and bactericidal effects from an air discharge plasma in contact with water: evidence for the formation of peroxynitrite through a pseudo-second-order post-discharge reaction of H_2O_2 and HNO_2. Plasma Sources Science & Technology **23**.

Müller K, Linkies A, Vreeburg RAM, Fry SC, Krieger-Liszkay A, Leubner-Metzger G. 2009. *In vivo* cell wall loosening by hydroxyl radicals during cress (*Lepidium sativum* L.) seed germination and elongation growth. Plant Physiology **150**, 1855-1865.

Nakabayashi K, Bartsch M, Xiang Y, Miatton E, Pellengahr S, Yano R, Seo M, Soppe WJ. 2012. The time required for dormancy release in Arabidopsis is determined by DELAY OF GERMINATION1 protein levels in freshly harvested seeds. The Plant Cell **24**, 2826-2838.

Nonogaki H. 2017. Seed biology updates - highlights and new discoveries in seed dormancy and germination research. Front Plant Sci **8**, 524.

Ogawa M, Hanada A, Yamauchi Y, Kuwahara A, Kamiya Y, Yamaguchi S. 2003. Gibberellin biosynthesis and response during *Arabidopsis* seed germination. The Plant Cell **15**, 1591-1604.

Sakiyama Y, Graves DB, Chang HW, Shimizu T, Morfill GE. 2012. Plasma chemistry model of surface microdischarge in humid air and dynamics of reactive neutral species. Journal of Physics D-Applied Physics **45**.

Sanchez-Montesino R, Bouza-Morcillo L, Marquez J, Ghita M, Duran-Nebreda S, Gomez L, Holdsworth MJ, Bassel G, Onate-Sanchez L. 2019. A regulatory module controlling GA-mediated endosperm cell expansion is critical for seed germination in Arabidopsis. Mol Plant **12**, 71-85.

Scheler C, Weitbrecht K, Pearce SP, Hampstead A, Buettner-Mainik A, Lee K, Voegele A, Oracz K, Dekkers B, Wang X, Wood A, Bentsink L, King J, Knox P, Holdsworth M, Muller K, Leubner-Metzger G. 2015. Promotion of testa rupture during garden cress germination involves seed compartment-specific expression and activity of pectin methylesterases. Plant Physiology **167**, 200-215.

Seo M, Aoki H, Koiwai H, Kamiya Y, Nambara E, Koshiba T. 2004. Comparative studies on the Arabidopsis aldehyde oxidase (AAO) gene family revealed a major role of AAO3 in ABA biosynthesis in seeds. Plant and Cell Physiology **45**, 1694-1703.

Steinbrecher T, Leubner-Metzger G. 2017. The biomechanics of seed germination. Journal of Experimental Botany **68**, 765-783.

Vasko CA, Liu DX, van Veldhuizen EM, Iza F, Bruggeman PJ. 2014. Hydrogen peroxide production in an atmospheric pressure RF glow discharge: Comparison of models and experiments. Plasma Chemistry and Plasma Processing **34**, 1081-1099.

Voegele A, Linkies A, Müller K, Leubner-Metzger G. 2011. Members of the gibberellin receptor gene family *GID1* (*GIBBERELLIN INSENSITIVE DWARF1*) play distinct roles during *Lepidium sativum* and *Arabidopsis thaliana* seed germination. Journal of Experimental Botany **62**, 5131-5147.

Winter D, Vinegar B, Nahal H, Ammar R, Wilson GV, Provart NJ. 2007. An "electronic fluorescent pictograph" browser for exploring and analyzing large-scale biological data sets. PLoS ONE **2**, e718.

Winter J, Tresp H, Hammer MU, Iseni S, Kupsch S, Schmidt-Bleker A, Wende K, Dunnbier M, Masur K, Weltmannan KD, Reuter S. 2014. Tracking plasma generated H_2O_2 from gas into liquid phase and revealing its dominant impact on human skin cells. Journal of Physics D-Applied Physics **47**.

Yan A, Wu MJ, Yan LM, Hu R, Ali I, Gan YB. 2014. AtEXP2 is involved in seed germination and abiotic stress response in Arabidopsis. PLoS ONE **9**.

Yan D, Easwaran V, Chau V, Okamoto M, Ierullo M, Kimura M, Endo A, Yano R, Pasha A, Gong Y, Bi YM, Provart N, Guttman D, Krapp A, Rothstein SJ, Nambara E. 2016. NIN-like protein 8 is a master regulator of nitrate-promoted seed germination in Arabidopsis. Nature Communications **7**, 13179.

Zhang Y, Chen B, Xu Z, Shi Z, Chen S, Huang X, Chen J, Wang X. 2014. Involvement of reactive oxygen species in endosperm cap weakening and embryo elongation growth during lettuce seed germination. Journal of Experimental Botany **65**, 3189-3200.
